# A Biomass-Inspired Hydrogel Patch for Intelligent Pain Monitoring and On-Demand Analgesia

**DOI:** 10.34133/research.1112

**Published:** 2026-02-11

**Authors:** Yibin Lin, Yan Wu, Haiting Fan, Yuling Wu, Hongcai Liang, Wenjing Lin, Liangtian Lan, Duoqu Chen, Jiaxin Li, Xia Feng, Shuai Zhao, Guobin Yi

**Affiliations:** ^1^ School of Chemical Engineering and Light Industry, Guangdong University of Technology, Guangzhou 510006, China.; ^2^ Department of Anesthesiology, First Affiliated Hospital, Sun Yat-sen University, Guangzhou 510080, China.; ^3^ The Second Affiliated Hospital of Guangzhou University of Chinese Medicine, Guangzhou 510120, China.; ^4^ Guangdong Provincial Laboratory of Chemistry and Fine Chemical Engineering Jieyang Center, Jieyang 515200, China.

## Abstract

The development of efficacious pain management strategies remains a pivotal challenge, requiring the creation of sustainable, biomass-derived interfaces for real-time techniques. Existing assessment approaches are either invasive, rendering them inappropriate for extended home-based monitoring, or dependent on patient-reported subjective evaluations. In this study, we fabricated a multifunctional biomass-inspired polydopamine-based hydrogel (polyvinyl alcohol [PVA]/polyacrylamide [PAM]/lithium chloride [LiCl]/polydopamine [PDA]/lidocaine hydrochloride [LiH]) wearable patch. Encapsulating lidocaine, a local anesthetic, this biomass-composite patch integrated pain-sensing-assisted assessment and treatment functionalities. It exhibited remarkable properties, including good stretchability (534.22%), low modulus (0.044 kPa), fine tissue adhesion (1.82 kPa), high conductivity (3.90 S m^−1^), rapid self-healing ability, and antibacterial properties. The patch enabled accurate sensing of diverse motion-related signals. Combined with deep learning algorithms, patients diagnosed with scapulohumeral periarthritis and lumbar diseases were recruited as volunteers for pain signal monitoring and evaluation (accuracy rate ~100%). Moreover, the hydrogel patch prolonged local photothermal analgesia in paw withdrawal threshold (>31% vs. Ctrl) and cumulative pain score (<10) by using a mouse plantar incision pain model. PVA/PAM/LiCl/PDA-based hydrogels elicited no detectable skin irritation or sensitization under the tested conditions. Therefore, this work not only pioneers the construction of a wearable integrated patch for pain management featuring “AI-assisted sensing evaluation” and “on-demand therapy”, but also provides a highly promising intelligent solution based on biomass-derived patches for the objective and prospective assessment and treatment of various types of pain.

## Introduction

The International Association for the Study of Pain defines pain as “an unpleasant sensory and emotional experience associated with or similar to actual or underlying tissues” [1,2], which can occur at any age with any disease. It affects the quality of daily life and work, including sensory abnormalities, somatic pain, mood disorders, and social withdrawal [3]. Data show that the number of pain sufferers in the global population continues to rise year after year and contributes consequentially to high morbidity, mortality, and disability, as well as burdens the health care system with large economic costs to the country [4,5]. In addition, longer treatments and therapeutic procedures impose a substantial financial burden on patients [6]. Current treatments for pain, especially oral opioid analgesics, are limited in their use and effectiveness and often cause side effects. Lidocaine and ropivacaine are commonly used clinically as amide-type medium and long-duration local anesthetics, which inhibit nerve signaling by blocking sodium channels and are characterized by the fast onset of action, good penetration, and strong diffusion. However, their subcutaneous effects do not last more than 4 h [7–9]. The typical acute pain is about 72 h, and chronic pain lasts longer. Long-lasting delivery systems for local anesthetics are new technologies being developed for pain, including hydrogel patches, injectable liquids, and injectable micro-nano level drug delivery systems [9–13]. However, frequent subcutaneous injections of local anesthetics can be associated with many operational inconveniences and increase the fear of patients [14].

In recent years, hydrogels have gradually become important candidates for drug delivery due to their unique advantages such as superior biocompatibility, mechanical properties, self-healing ability, and adhesion [15–19]. Loading local anesthetics into hydrogel patches can not only release local anesthetics slowly, prolong the half-life, and enhance the anesthetic effect, but also reduce the dosage of local anesthetics and avoid fluctuations in blood concentration caused by multiple administrations [20,21]. For example, our group prepared a polydopamine-based hydrogel patch in 2024, which is superior to the commercial lidocaine patch in terms of lidocaine release and analgesic effect [22]. In addition, with the rapid development of emerging flexible electronics technology, a new generation of hydrogel patches forming various flexible sensors for smart monitor, integrated assessment, and treatment have been developed to realize in situ monitoring and on-demand treatment of diseases [23–29]. For example, Roy et al. [30] synthesized a multifunctional conductive β-CD-g-(pAAm/pAETAc) hydrogel and demonstrated its effectiveness in monitoring motor dysfunction in conditions simulating Parkinson’s disease. Yang et al. [31] developed an adhesive skin patch containing polyaspartic acid-modified dopamine/ethyl-based ionic liquid hydrogel as a stimulation/recording device to capture electromyographic signals to diagnose peripheral neuropathy of sensory, motor, and mixed nerves in clinical cases.

Composed of natural polyphenolic tannins (TA) and phenylboronic acid grafted hyaluronic acid (HA-PBA), Wang et al. [32] introduced a new MXene/HA-PBA/TA smart hydrogel that could be used in many applications such as monitoring human health, recording of tiny electrical signals on the skin, photothermal therapy, and hemostasis. Smart wound diagnostic and therapeutic dressings can not only realize real-time monitoring of wound status through various biomarkers (including pH, temperature, and uric acid), but also deliver drugs through mechanical response, near-infrared triggering, or ultraviolet response to treat wounds [33–36]. Therefore, multifunctional hydrogel patches integrating pain assessment and treatment are of great significance for the smart pain management. However, to date, only a limited number of literature reports have focused on hydrogel patches for analgesia or pain assessment, and no studies have been documented on such multifunctional integrated hydrogel patches.

**Fig. 1.**
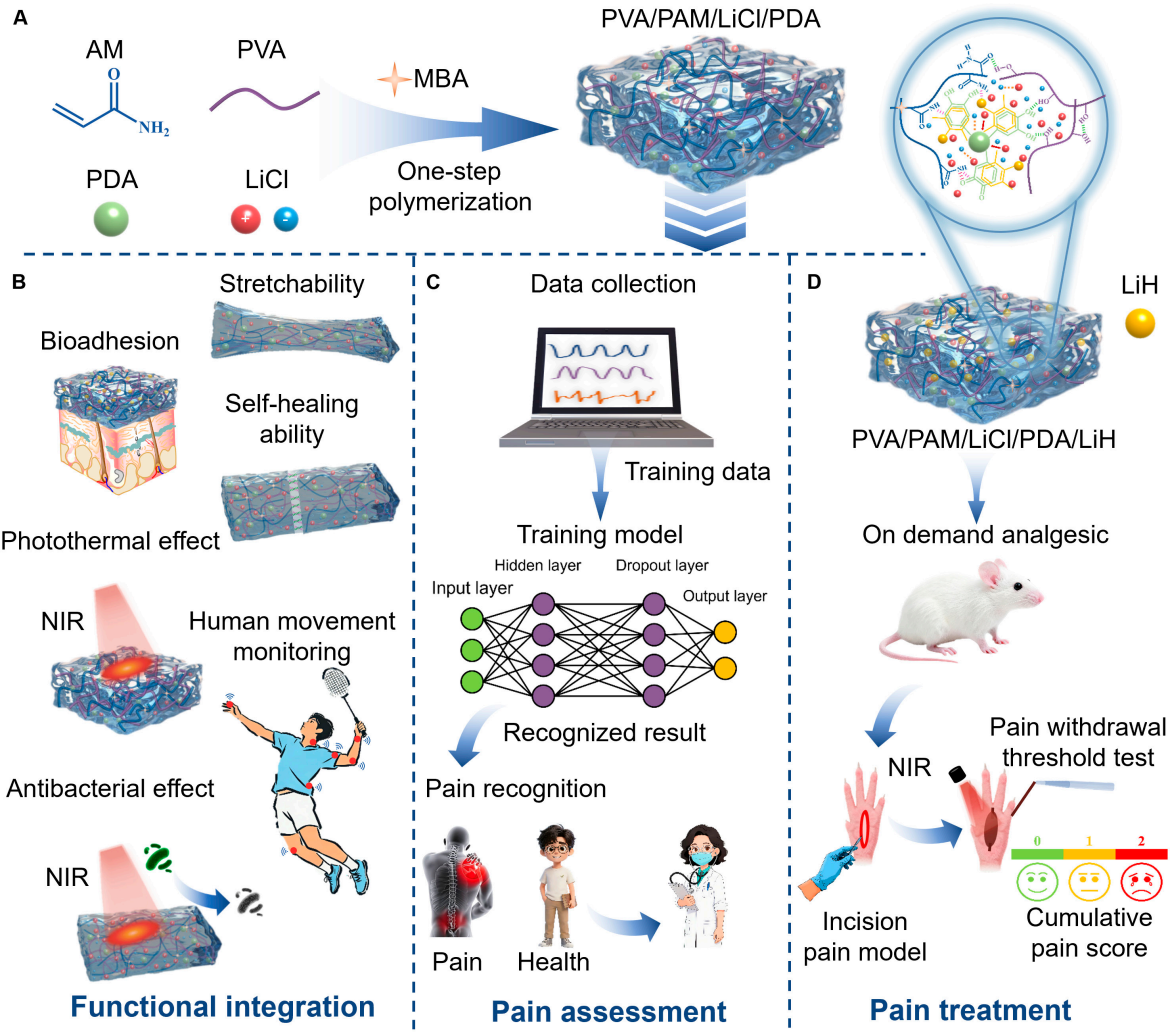
(A) Schematic for the preparation of stretchable, bioadhesive, conductive, and photothermal PVA/PAM/LiCl/PDA hydrogel. (B) Functional integration of PVA/PAM/LiH/PDA hydrogel in terms of stretchability, bioadhesion, photothermal effect, self-healing ability, antibacterial effect, and human motion monitoring as well as its applications in (C) pain assessment and (D) treatment.

Herein, we first rationally designed and fabricated a multifunctional biomass-inspired conductive hydrogel patch (PVA/PAM/LiCl/PDA) encapsulating the local anesthetic lidocaine hydrochloride (LiH) for integrated pain assessment and treatment (Fig. 1). This patch synthesized via a one-pot reaction using polyacrylamide (PAM), polyvinyl alcohol (PVA), polydopamine (PDA), and ionic conductor LiCl. PAM and PVA enhanced adhesiveness. Biomass-derived PDA improved adhesion, photothermal conversion, photothermal antibacterial activity, and on-demand photothermal analgesia. LiCl boosted hydrogel antifreezing, water retention, and ionic conductivity for sensing. Via tuning component ratios, we explored tunable mechanical, adhesive, conductive properties and self-healing capability. We further explored its application as a flexible wearable sensor for smart sensing including detecting clinical motion-related signals and applying deep learning to recognize signals from pain disorders (frozen shoulder, lumbar disc herniation, and lumbar facet joint dysfunction). We then assessed hydrogel cytotoxicity using mouse macrophage (Raw 264.7) cells and human stem fibroblast (HSF) cells, and antimicrobial properties using gram-negative *Escherichia coli* and gram-positive *Staphylococcus aureus*. Using a mouse plantar incision pain model, we evaluated the patch’s localized photothermal on-demand long-lasting analgesic effect in paw withdrawal threshold (PWT) and cumulative pain score (CPS). Finally, we assessed the skin contact safety of the hydrogel. Overall, this lidocaine-loaded biomass hydrogel sensor enables artificial intelligence-assisted pain assessment and treatment, thereby providing an innovative solution for real-time medical monitoring and intelligent pain disorder management.

## Results and Discussion

### Formation, structure characterization, and mechanical properties of hydrogels

To enable smart pain monitoring and therapy, we designed multifunctional hydrogels with stretchability, low modulus, suitable bioadhesion, and high conductivity. Uniform PDA NPs (Fig. S1) were mixed with PVA, acrylamide (AM), LiCl, and N,N′-methylenebisacrylamide (MBA), forming a double PVA/PAM/LiCl/PDA hydrogel network. PDA enhanced photothermal conversion (Fig. S2), antibacterial activity, and on-demand analgesia. LiCl improved antifreeze and sensing conductivity.

**Fig. 2.**
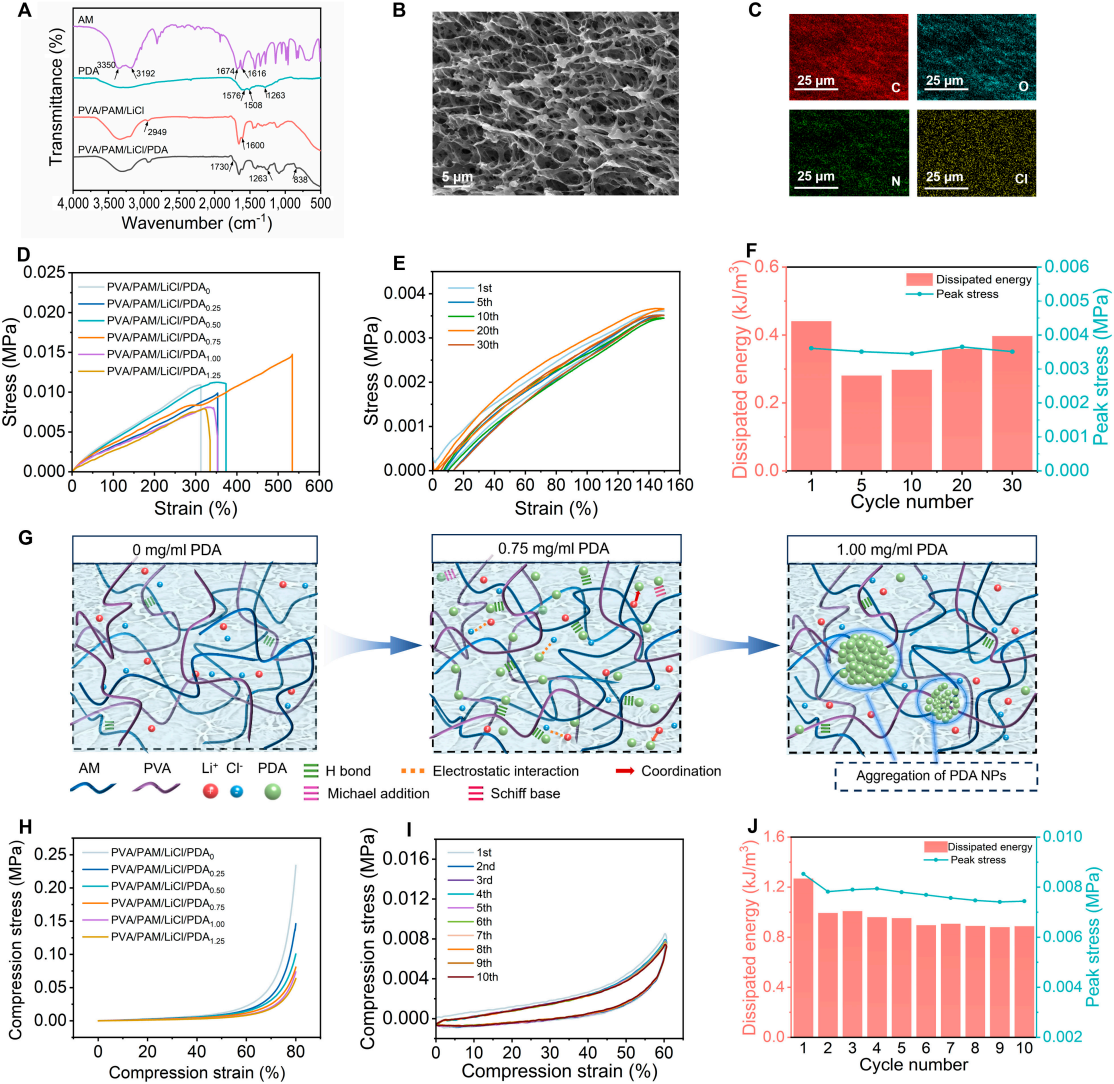
Characterization and mechanical properties of the PVA/PAM/LiCl/PDA hydrogel. (A) FT-IR spectra of AM, PDA, PVA/PAM/LiCl, and PVA/PAM/LiCl/PDA. (B) SEM image of the PVA/PAM/LiCl/PDA hydrogel. (C) Element mappings of C, O, N, and Cl. (D) Tensile stress–strain curves of the PVA/PAM/LiCl/PDA hydrogel with different PDA NP contents. (E) Loading–unloading curves of PVA/PAM/LiCl/PDA_0.75_ hydrogel at 150% strain for 30 cycles and (F) corresponding dissipated energy and peak stress. (G) Schematic illustrations of the PVA/PAM/PDA hydrogel networks with PDA concentrations of 0, 0.75, and 1.00 mg/ml, respectively. (H) Compression stress–strain curves of the PVA/PAM/LiCl/PDA hydrogel with different PDA NPs contents. (I) Loading–unloading curves of PVA/PAM/LiCl/PDA_0.75_ hydrogel at 60% compression strain for 10 cycles and (J) corresponding dissipated energy and peak stress.

As shown in Fig. 2A, the peaks of AM at 3,350, 3,192, 1,616, and 1,674 cm^−1^ were attributed to the stretching vibrations of N-H, C-H, C=C, and C=O, respectively. PDA exhibited overlapping O-H/N-H peaks (3,000 to 3,500 cm^−1^), phenolic C-O stretch peaks (1,263 cm^−1^), aromatic C=C stretch peaks (1,508 and 1,576 cm^−1^), and aromatic C-H bending peaks (874 cm^−1^). For the PVA/PAM/LiCl hydrogel, broad and narrow peaks of O-H and N-H appeared around the peak 3,000 to 3,500 cm^−1^; 2,949 cm^−1^ was the stretching vibration attributed to C-H. The absence of the C=C peaks at 1,600 cm^−1^ confirmed polymerization. PVA/PAM/LiCl/PDA hydrogel displayed broader 3,000 to 3,500 cm^−1^ peaks (catechol-induced intermolecular H-bonds) and a widened 1,730 cm^−1^ peak (C=O stretch and N-H bending). PDA-specific peaks at 1,263 and 838 cm^−1^ belonged to phenolic C-O and aromatic C-H verified its incorporation. Lyophilized PVA/PAM/LiCl/PDA had a 3D interconnected porous structure (Fig. 2B), with homogeneous distribution of C, N, O, and Cl elements, indicating a uniform distribution of chemical bonding and physical interactions within the hydrogel (Fig. 2C).

Hydrogel patch mechanics were tuned via PDA NP content. Increasing PDA NPs from 0 to 0.75 mg ml^−1^ elevated hydrogel elongation at break (312.33% to 534.22%) and toughness (18.77 to 40.84 kJ m^−3^) (Fig. 2D), while further increasing to 1.25 mg ml^−1^ reduced these to 334.88% and 14.19 kJ m^−3^, respectively. Appropriate PDA NP loading enabled catechol/quinone groups to form dynamic interactions (hydrogen bonds, electrostatic interaction, metal coordination, Schiff-base reactions, and Michael additions) with the hydroxyl groups in PVA and the amide groups in PAM and LiCl, facilitating energy dissipation to enhance stretchability and toughness. Excessive PDA NPs induce aggregation within the hydrogel network, impairing mechanical performance, resulting in decreased mechanical performance (Fig. 2G).

In both tensile and compression tests (Fig. 2H and Figs. S3 and S4), the PVA/PAM/LiCl/PDA hydrogels exhibited low tensile modulus (0.03 to 0.06 kPa) and compression modulus (0.06 to 0.11 kPa), which could reduce the discomfort from mechanical mismatch during wear and enhance strain sensing sensitivity. Cyclic tensile (Fig. 2E) and compression (Fig. 2I) tests evaluated fatigue resistance. Hydrogels rapidly recovered their original state in cyclic compression (Fig. S5). After the first compression and stretch loading–unloading, there was a more decrease in dissipation energy, which was attributed to the inability to recover instantaneously from the noncovalent interactions in the hydrogel network. Beyond the first cycle, stable dissipation energy, maximum stress, and overlapping hysteresis loops confirmed good fatigue resistance and elasticity (Fig. 2F and J). Increasing PDA NP content reduced tensile and compression moduli, likely due to antioxidant PDA consuming APS-derived free radicals to inhibit free-radical polymerization [37], thus enabling tunable stretchability and low modulus. Wide-range cyclic tensile and compression tests assessed PVA/PAM/LiCl/PDA hydrogel adaptability to deformation. It showed superior elastic recovery at 10% to 400% tensile strain (Figs. S6 and S7) and good reversible deformation at 10% to 65% compression strain (Fig. S8), with compression energy dissipation positively correlating with strain (Fig. S9), indicating that the PVA/PAM/LiCl/PDA hydrogel maintained stable mechanical response properties while absorbing energy.

### Adhesive properties of hydrogels

**Fig. 3.**
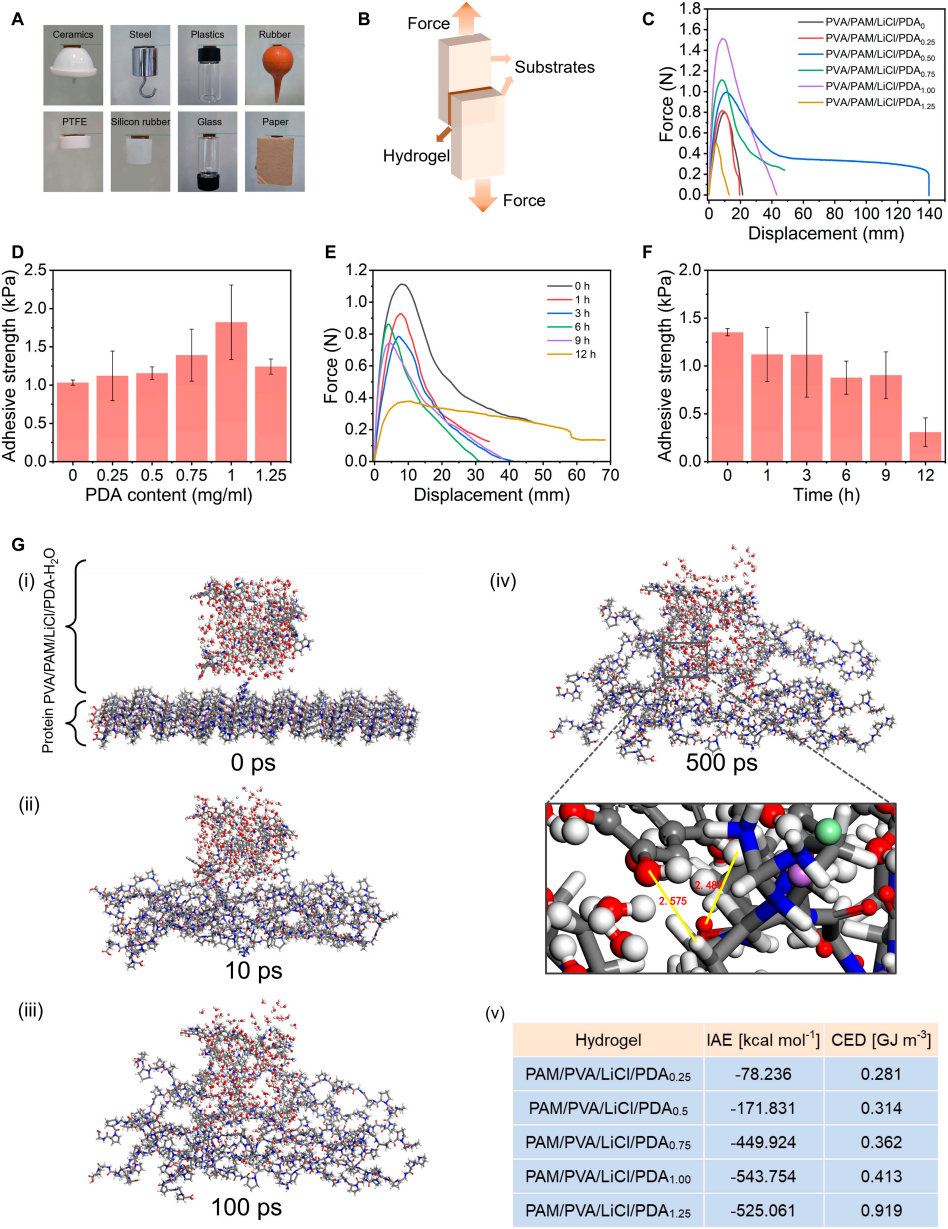
Adhesive properties and MD simulation of the PVA/PAM/LiCl/PDA hydrogel. (A) Adhesion of the PVA/PAM/LiCl/PDA_0.75_ hydrogel to various substrates. (B) Schematic of lap shear tests. (C) Force–displacement curves and (D) adhesion strength of the PVA/PAM/LiCl/PDA hydrogel. (E) Force–displacement curves and (F) adhesion strength of PVA/PAM/LiCl/PDA_0.75_ hydrogel loadings on porcine skin at different adhesion durations. (G) MD simulation of the adhesion process of the PAM/PVA/LiCl/PDA hydrogel on a skin surface: (i) initial state (0 ps); (ii) intermediate state (10 ps); (iii) intermediate state (100 ps); (iv) final state (500 ps); and (v) calculated IAE and CED values of different hydrogels on protein surfaces.

As epidermal sensors, hydrogels required skin-mimetic mechanical properties and strong skin adhesion to ensure signal detection stability and sensitivity. The PVA/PAM/LiCl/PDA_0.75_ hydrogel patches adhered well to diverse substrates, including crucibles, weights, plastics, glass, rubber, polytetrafluoroethylene, silicone, and paper (Fig. 3A and Movie S1), driven by catechol groups in PDA, hydroxyl groups in PVA, and amide groups in PAM interacting with substrate interfacial functional groups. Lap shear tests on fresh porcine skin in Fig. 3B showed that hydrogel adhesion strength first increased then decreased as the PDA content was raised from 0 to 1.25 mg ml^−1^, with values rising from 1.03 to 1.82 kPa, then falling to 1.24 kPa (Fig. 3C and D). PDA content was fixed at 0.75 mg ml^−1^ to balance tensile properties, low modulus, and adhesion. Adhesion strength decreased gradually with prolonged duration (Fig. 3E and F), but remained 0.90 kPa after 9 h on porcine skin, sufficient for reliable sensing and efficient transdermal drug delivery.

To date, molecular dynamics (MD) simulations have been limited to simulate the interactions of hydrogel with glass and polypropylene substrates [15,16], and no reports on MD simulations of the interaction between hydrogel patches and the skin interface have been documented. In this work, we pioneered the use of MD simulations to investigate the hydrogel patch–skin interface interaction. Specifically, MD simulations further explored PDA content effects on hydrogel adhesion, using 5 PAM/PVA/LiCl/PDA systems (0.25 to 1.25 mg ml^−1^ PDA) to mimic interactions with human epidermis (type I collagen, >80% skin content) (Figs. S10 and S11). As shown in Fig. 3G, the interface between the PAM/PVA/LiCl/PDA_1.00_ hydrogel layer and the protein layer was separated at 0 ps. However, as time passed, the interface of the 2 layers started to interact at 10 ps. After 100 ps of simulation, the degree of aggregation of the 2 interfaces increased and reached the final stable result at 500 ps. The distance between the hydrogel and the protein was in the range of 1.900 to 2.600 Å, which corresponded to the typical length range for hydrogen bonds [38,39].

Cohesive energy density (CED), a measure of intermolecular interaction energy per unit volume, increased as the PDA content increased (Fig. 3G and Table S1), confirming tunable hydrogel intermolecular interactions. We further evaluated interfacial adhesion energy (IAE), a measure of hydrogel-protein adhesion strength. As concentration of PDA increased to 1 mg ml^−1^, the interfacial adhesion strength increased, reaching the highest IAE value of −543.754 kcal mol^−1^. However, the interfacial adhesion strength decreased to −525.061 kcal mol^−1^ when the PDA concentration increased to 1.25 mg ml^−1^. This series of interfacial adhesion performance simulation calculations was consistent with our experimental results. In summary, both experiment and MD simulations confirmed that PDA content could modulate PAM/PVA/LiCl/PDA hydrogel–skin interfacial adhesion and revealed the underlying mechanism.

### Self-healing, antifreezing, and sensing properties of hydrogels

**Fig. 4.**
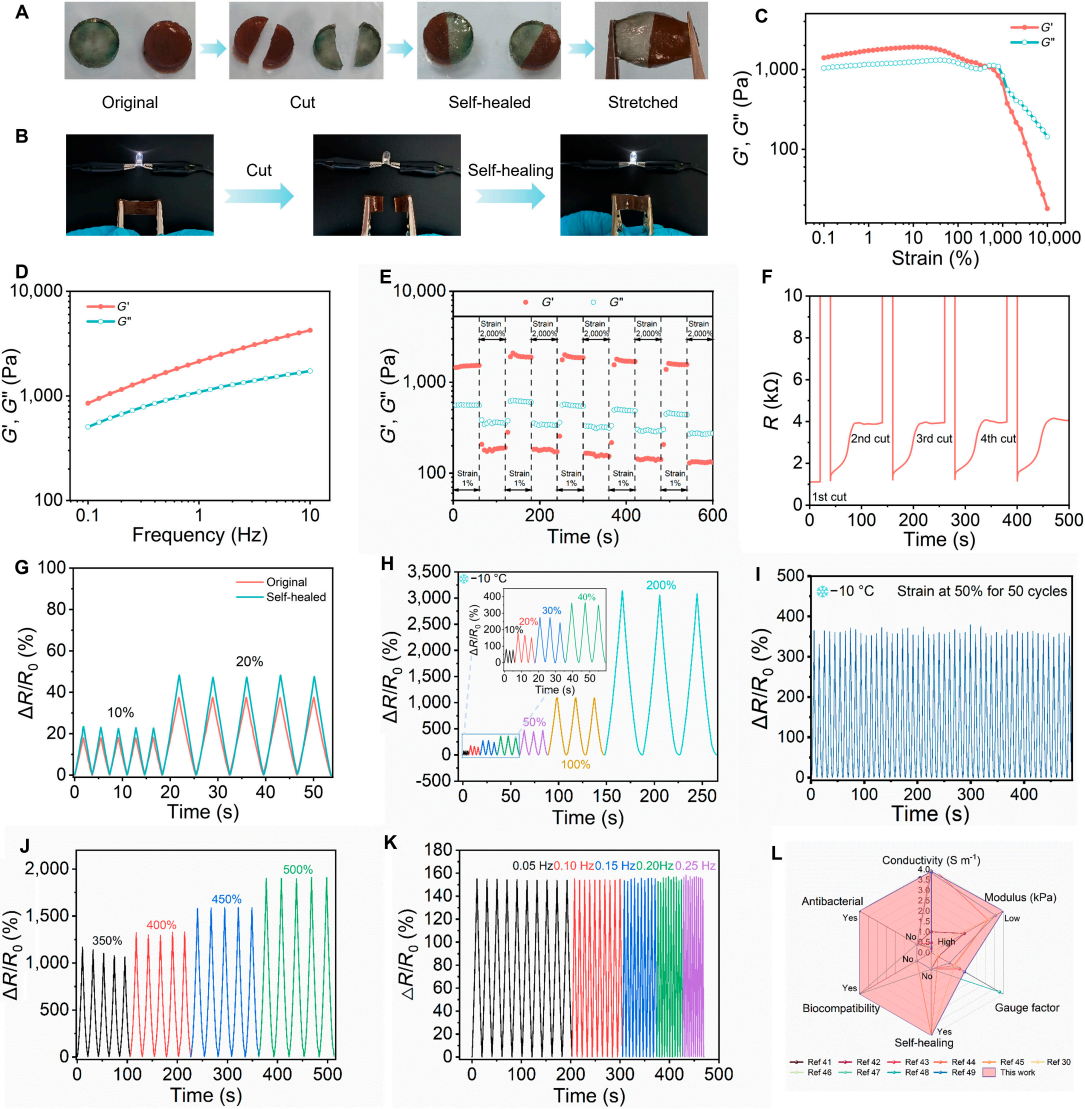
Self-healing, antifreezing, and sensing properties of the PVA/PAM/LiCl/PDA_0.75_ hydrogels. (A) Self-healing demonstration process. (B) Illuminance variation of the LED bulb during the cutting and healing process. (C) Strain sweep, (D) frequency sweep, and (E) dynamic step-strain sweep. (F) Resistance changes during the cutting and healing cycles. (G) Relative resistance variation of the original and self-healing hydrogel. (H) Relative resistance variation of the hydrogel with different strains at −10 °C. (I) Sensing stability test of hydrogel strain sensor at a strain of 50% for 50 tensile cycles (−10 °C). (J) Relative resistance variation with 350% to 500% strains. (K) Relative resistance variation with different frequencies. (L) Comparison of PVA/PAM/LiCl/PDA hydrogel strain sensors with previously reported sensors.

Sensors possessing self-healing properties enhance performance stability and prolong their operational lifespan. Mechanical/electrical self-healing of hydrogels was first carried out via cut-healing tests (4 h, 26 °C, 60% relative humidity) showing tension tolerance (Fig. 4A and Movie S2) and LED circuit integration showing extinguishing on cutting and brightening on reconnection (Fig. 4B). Quantitative studies of the hydrogels’ self-healing properties were then performed. Strain sweep tests indicated that the gel point of the PVA/PAM/LiCl/PDA hydrogel was approximately 500% (Fig. 4C). Frequency sweep results (Fig. 4D) showed that storage modulus (*G*′) was consistently greater than loss modulus (*G*″) at frequencies ranging from 0.1 to 10 Hz and a strain of 1%, indicating good elasticity and structural stability of the PVA/PAM/LiCl/PDA hydrogel. Dynamic step-strain sweep results (Fig. 4E) revealed that when high strain (2,000%) was applied, the value of *G*′ dropped below *G*″, indicating the structural collapse of the hydrogel. Once the applied dynamic strain was restored to 1%, the value of *G*′ sharply increased and immediately exceeded *G*″, indicating recovery to the hydrogel state. Moreover, both *G*′ and *G*″ could fully recover rapidly. This process was repeatable even after 5 alternating cycles, demonstrating the good self-healing capability of the PVA/PAM/LiCl/PDA hydrogel. Resistance changes during cutting/healing were quantified with spiking to near infinity on cutting and recovering in minutes (Fig. 4F). Sensor tests in Fig. 4G showed posthealing (25°C, 48 h) signal recovery.

The impact of LiCl on the antifreezing properties of PVA/PAM/PDA hydrogels was systematically assessed via low-temperature experiments. At −10 °C, the PVA/PAM/PDA hydrogel without LiCl exhibited whitening and hardening (Fig. S12A and E), indicating that it was prone to freezing and lost its flexibility and functionality at low temperatures. In contrast, the PVA/PAM/LiCl/PDA hydrogel maintained high flexibility (Fig. S12B) and could withstand complex deformations (Fig. S12C, D, and F), including bending, twisting, and stretching. Cyclic tensile tests further quantified their retained excellent mechanical properties, wide strain sensing range (10% to 200%), and stable sensing output (Fig. 4H). Specifically, after 50 cycles of tensile testing at 50% strain, the hydrogel showed no mechanical/electrical degradation (Fig. 4I and Fig. S13), indicating excellent fatigue resistance.

High-conductivity hydrogels are indispensable for highly sensitive strain sensors. LiCl significantly enhanced PVA/PAM/LiCl/PDA hydrogel conductivity. The introduction of PDA slightly increased the conductivity of hydrogels from 3.38 to 3.90 S m^−1^ (Fig. S14) aided by phenolic quinone–Li^+^ redox pairs [40]. The hydrogel patch’s strain sensing capability was preliminarily validated via series-connected green LED with LED lit initially, and dimmed on stretching due to increased resistance (Fig. S15A). The gauge factors (GF) at different strain response intervals were 1.75 (0% to 50%), 3.45 (50% to 350%), and 5.31 (350% to 500%) (Fig. S15B). Subsequently, the hydrogel patch’s sensing response to diverse strains and frequencies was investigated. Electrical signals from 5 stretching–unloading cycles at the same strain remained consistent, indicating excellent sensitivity and reversibility (Fig. S15C to E and Fig. 4J). At 0.05 to 0.25 Hz, signal amplitude was largely unchanged (Fig. 4K). Furthermore, the hydrogel exhibited a relatively short response time (172 ms) and recovery time (189 ms), and maintains good stability even after multiple cycles (Fig. S16). After 500 cycles of stretching at 50% strain, the electrical signals of the hydrogel patch showed no obvious attenuation or amplification, confirming its good fatigue resistance and cyclic stability (Fig. S17). To highlight the outstanding overall performance of the PVA/PAM/LiCl/PDA hydrogel strain sensor, based on certain adhesiveness, we compared it with previously reported hydrogel sensors in terms of conductivity, modulus, sensitivity, self-healing ability, biocompatibility, and antibacterial performance (Fig. 4L and Table S2) [30,41–49]. It is evident that the PVA/PAM/LiCl/PDA hydrogel strain sensor surpassed others in all these aspects, demonstrating its great potential for applications in flexible wearable devices.

### Hydrogel applications for monitoring human movements

**Fig. 5.**
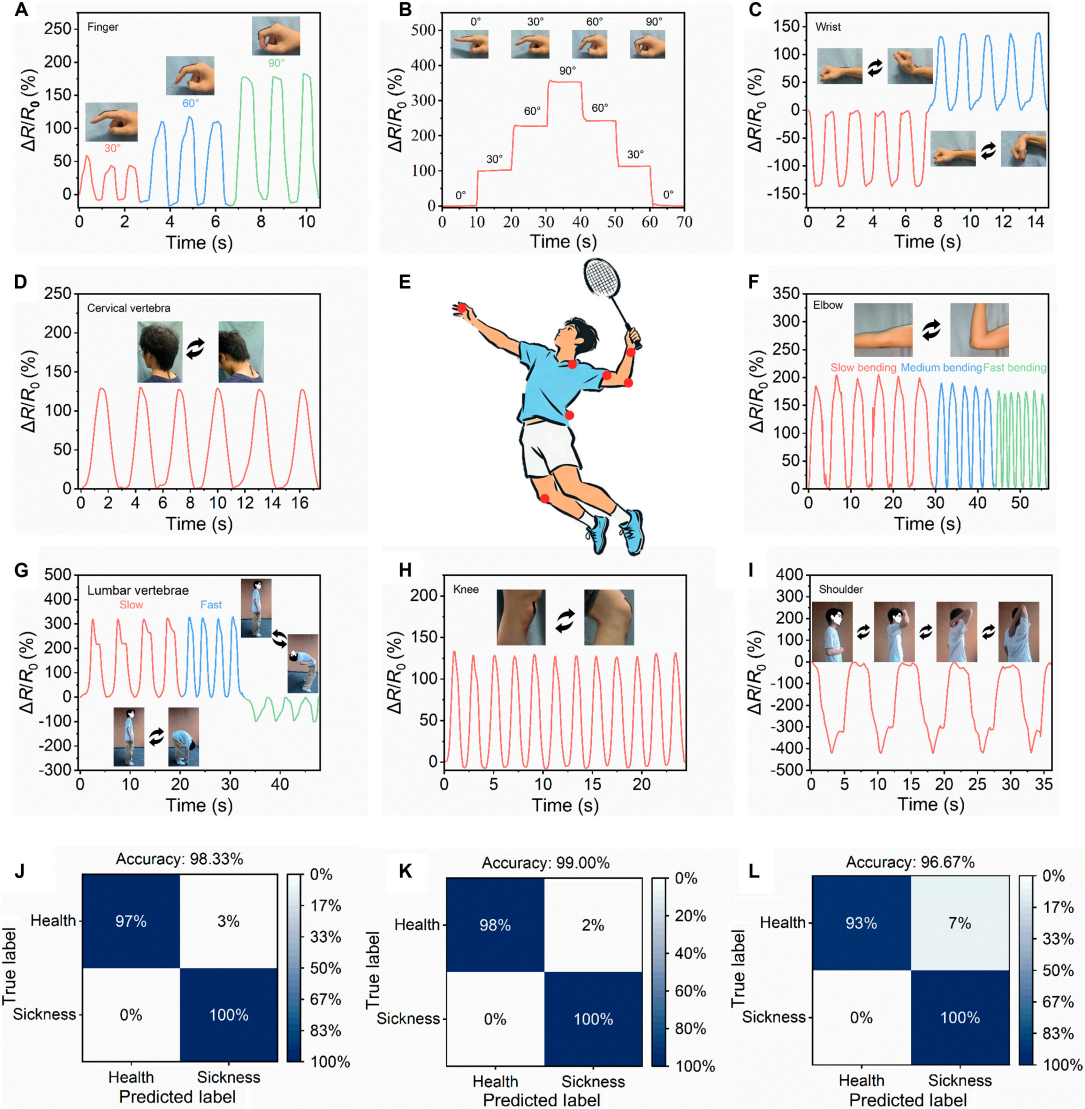
PVA/PAM/LiCl/PDA hydrogel applications for monitoring human movements. Real-time detection signals during human movement, including flexion and extension of (A and B) finger, (C) wrist, (D) cervical vertebrae, (F) elbow, (G) lumbar vertebrae, and (H) knee, as well as (I) abduction, internal rotation, and external rotation of the shoulder joint. (E) Schematic of sensing sites. The confusion matrix for identification (J) shoulder motion, (K) lumbar flexion, and (L) lumbar extension disorders.

The hydrogel sensors were strategically positioned on joints (fingers, shoulders, elbows, wrists, and knees) to effectively track dynamic motions (flexion, extension, adduction, abduction, and internal/external rotation) and real-time angles (Fig. 5E). For example, bending a finger from 0° to 90° and then returning it to the initial position produced corresponding changes in the electrical signal (Fig. 5A and B). The sensor then captured signals for wrist, cervical, elbow, lumbar vertebrae, and knee flexion or extension (Fig. 5C, D, F, G, and H), and reflected shoulder rotation or forward flexion (Fig. 5I).

Degenerative osteoarthritis causes pain and motor dysfunction, but lacks an accurate objective assessment method. PVA/PAM/LiCl/PDA hydrogel sensors integrated into volunteers’ shoulders and lumbar spine enabled real-time detection. Via standardized sequences (internal/external rotation and flexion), individuals with a healthy shoulder showed smooth cycles with stable signals (Fig. S18A), while volunteers with a frozen shoulder or shoulder girdle abnormalities had incomplete cycles, pain, and abnormal signals with smaller amplitude and frequent fluctuations (Fig. S18D). Similarly, lumbar disc herniation caused pain-limited flexion with lower limb radiating pain, numbness, and sensory/muscle deficits. Lumbar facet joint dysfunction was characterized by low back pain and restricted extension. As shown in Fig. S18B and C, and E and F, healthy volunteers had high-frequency signals, whereas patients showed low-frequency and low-amplitude signals, reflecting their limited range of motion and pain conditions. A deep learning system intelligently identified movement data from PVA/PAM/LiCl/PDA strain sensors to predict target area health.

Normal or abnormal movement states had distinct electrical signal waveforms (differential intensity or frequency) for distinction. The dataset was split into training, validation, and test sets at 1:1:1 for model robustness. After training, the overall recognition accuracy of normal or abnormal movements approached 100% (Fig. 5J to L). In summary, the hydrogel sensor adhered to skin and monitored subtle joint changes in real time. When combined with deep learning, the system accurately captured movement characteristics, offering objective, quantitative, and scientific clinical decision support and demonstrating crucial promise for future medical diagnostics.

### Photothermal, drug release, antibacterial, and analgesic properties of hydrogels

**Fig. 6.**
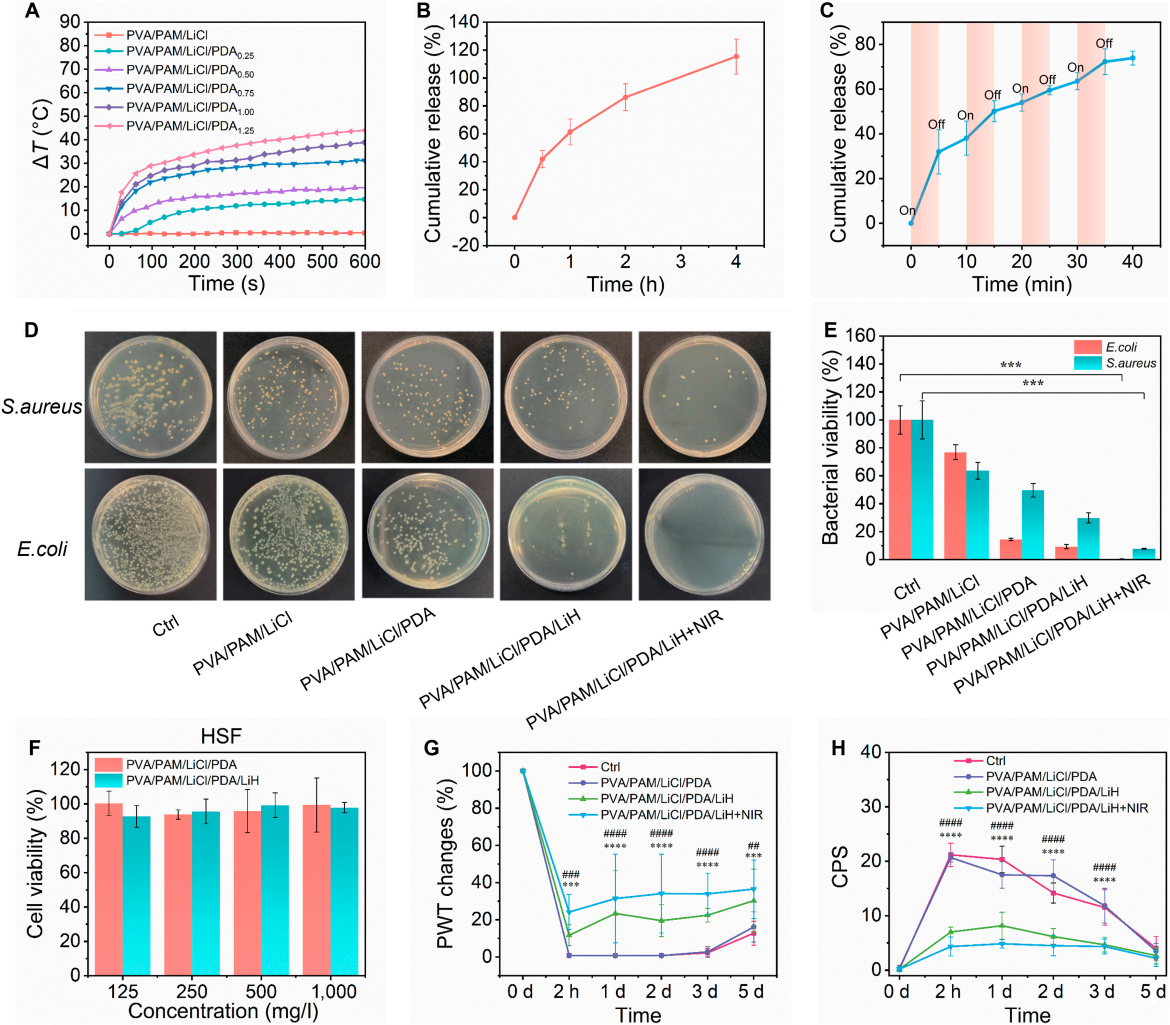
Photothermal, drug release, antibacterial, and analgesic properties of hydrogels. (A) Photothermal temperature rise curves under 808-nm NIR laser irradiation with different contents of PDA NPs. In vitro release of LiH from (B) the PVA/PAM/LiCl/PDA hydrogel and (C) PVA/PAM/LiCl/PDA+NIR. (D) Optical images of bacteria formed by *E. coli* and *S. aureus* treated with PVA/PAM/LiCl, PVA/PAM/LiCl/PDA, PVA/PAM/LiCl/PDA/LiH, and PVA/PAM/LiCl/PDA/LiH+NIR (808 nm, 0.30 W cm^−2^, 10 min). (E) Bacterial viabilities of *E. coli* and *S. aureus*. (F) Relative viability of HSF cells incubated with extracts of various concentrations of the PVA/PAM/LiCl/PDA and PVA/PAM/LiCl/PDA/LiH hydrogels after 24 h. (G) Changes in PWT of mice in each group compared to baseline. (H) The actual measurement value of CPS of mice. All values were expressed as the mean ± SD (*n* = 6). ***P < 0.001 and ****P < 0.0001 between PVA/PAM/LiCl/PDA/LiH+NIR and Ctrl at different times; ##*P* < 0.01, ###*P* < 0.001, and ####*P* < 0.0001 between PVA/PAM/LiCl/PDA/LiH+NIR and PVA/PAM/LiCl/PDA at different times.

PDA shows broad NIR (near infrared) absorption for efficient photothermal conversion, which enables PVA/PAM/LiCl/PDA hydrogel patch on-demand photothermal-controlled drug release for precise therapeutics. Their photothermal performance was highly tunable via adjusting PDA content and NIR laser power. Specifically, when PDA increased from 0 to 1.25 mg ml^−1^, the temperature rise (Δ*T*) increased from 0.39 to 43.981 °C after 10 min of NIR exposure with 0.30 W cm^−2^ power (Fig. 6A). Additionally, for the PVA/PAM/LiCl/PDA_0.75_ hydrogel patch, 5 min NIR (0.10 to 0.30 W cm^−2^) raised Δ*T* from 5.697 to 20.142 °C (Fig. S19). Its photothermal effect linearly correlated with NIR power (Fig. S21), and the Δ*T* remained above 34 °C after 3 cycles, demonstrating its excellent photothermal stability (Fig. S20). The photothermal conversion efficiency was 72.9%, calculated from the temperature-time cooling curve (Fig. S22) and Eqs. S6 to S8 of the Supplementary Materials. The ability of the PVA/PAM/LiCl/PDA/LiH hydrogel patches to release LiH was evaluated using an in vitro simulated drug release assay (Fig. 6B). The LiH release profile corresponded to the “on” and “off” cycles of NIR irradiation, increasing and decreasing synchronously with the induced temperature changes. Four cycles achieved 73.91% cumulative release in 30 min from PVA/PAM/LiCl/PDA hydrogel patches (Fig. 6C).

The antibacterial properties of the hydrogel patches were evaluated against *E. coli* and *S. aureus* using the plate counting method (Fig. 6D and E). Compared to the control, the antibacterial effect of PVA/PAM/LiCl was limited relative to PVA/PAM/PDA. In contrast, the viabilities of *E. coli* and *S. aureus* were significantly reduced in PVA/PAM/LiCl/PDA and PVA/PAM/LiCl/PDA/LiH. The antibacterial efficacy of PVA/PAM/LiCl/LiH was enhanced under NIR irradiation, potentially due to the synergistic effects of photothermal antibacterial capacities PDA-LiCl, and the rapid release of LiH.

We then conducted cytotoxicity tests on RAW 264.7 and HSF cells to evaluate the biocompatibility of hydrogel patches using the Cell Counting Kit-8 (CCK-8) assay. After 24-h coculture, the cell viability of both HSF and RAW 264.7 cell lines remained above 80% (Fig. 6F and Fig. S23). The effects of the hydrogels on cell morphology and proliferation were examined using Actin-4′,6-diamidino-2-phenylindole (DAPI) and Tubulin-DAPI immunofluorescence staining. Tubulin staining showed that HSF cells had typical spindle shapes with PVA/PAM/LiCl/PDA and PVA/PAM/LiCl/PDA/LiH, while no obvious differences in fluorescence intensity. Actin-DAPI costaining confirmed neither formulation affected the morphology or proliferation of HSF and RAW 264.7 cells, consistent with the CCK-8 assay (Fig. S24A to C).

Using a mouse plantar incision model, we incorporated LiH into PVA/PAM/LiCl/PDA to fabricate analgesic formulations. PWT and CPS were used to quantify pain behavior and analgesia. PWT was measured using Von Frey filaments applied via the up–down method, with all values normalized to the preoperative baseline (set as 100%). Postoperatively, Ctrl and PVA/PAM/LiCl/PDA groups showed PWT changes of 0.76% and 0.75%, indicating incision-induced mechanical allodynia and no analgesic effect of PVA/PAM/LiCl/PDA (Fig. 6G). Conversely, incorporation of the analgesic LiH yielded mechanical threshold elevations of 11.68% ± 5.59% (PVA/PAM/LiCl/PDA/LiH) and 24.14% ± 9.41% (PVA/PAM/LiCl/PDA/LiH+NIR), both significantly exceeding Ctrl and PVA/PAM/LiCl/PDA groups. NIR-induced local hyperthermia accelerated LiH release from the hydrogel, enhancing targeted analgesia. Over the subsequent 5 days, the changes in mechanical threshold in the PVA/PAM/LiCl/PDA/LiH and PVA/PAM/LiCl/PDA/LiH+NIR groups remained consistently higher than 19% and 31%, respectively. As shown in Fig. 6H, the CPS values of the PVA/PAM/LiCl/PDA/LiH group and the PVA/PAM/LiCl/PDA/LiH+NIR group remained relatively low (<10), demonstrating effective pain relief. In contrast, the CPS values in the PVA/PAM/LiCl/PDA group did not show any analgesic effects compared to the control group. These results suggested that the hydrogel patch has great potential as a smart device for NIR-enhanced, on-demand long-term local analgesia.

### Skin contact safety assessment of hydrogels

**Fig. 7.**
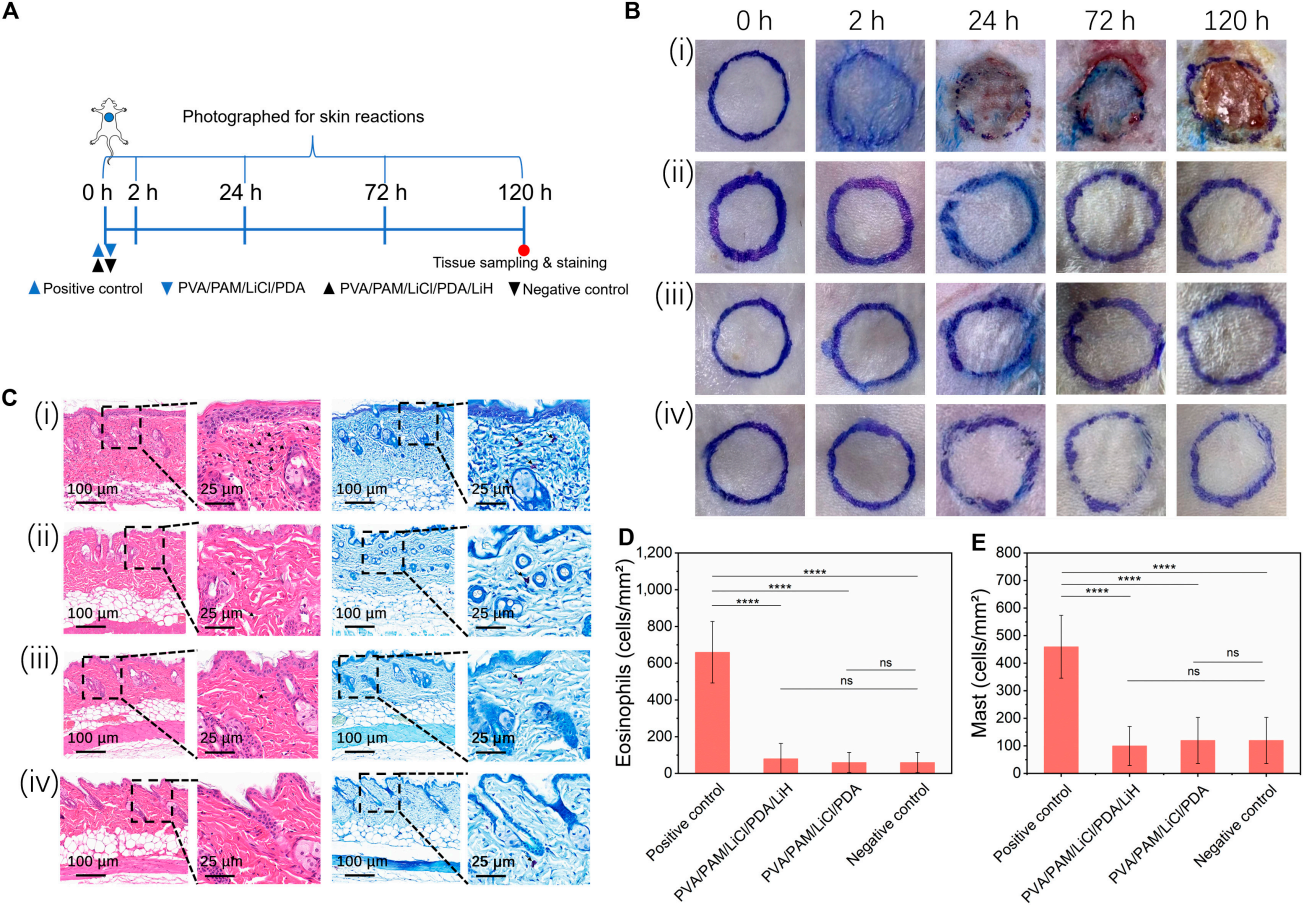
Skin contact safety assessment of hydrogels. (A) Schematic timeline of hydrogel application and observation time points. (B) Representative macroscopic photographs of skin sites from the 4 experimental groups: (i) positive control, (ii) PVA/PAM/LiCl/PDA/LiH, (iii) PVA/PAM/LiCl/PDA, and (iv) negative control. Blue circles indicated the 7-mm hydrogel application areas. (C) Representative images of H&E staining of (i) positive control, (ii) PVA/PAM/LiCl/PDA/LiH, (iii) PVA/PAM/LiCl/PDA, and (iv) negative control. Black arrows in the H&E-stained sections indicated eosinophils, and black arrows in the toluidine blue-stained sections denoted mast cells. Quantitative analysis of inflammatory cell infiltration; (D) eosinophil and (E) mast cell counts per high-power field (HPF) were presented as mean ± SD (*n* = 5), ****P < 0.0001 compared to the positive control group.

Local skin irritation/sensitization was evaluated for 120 h after hydrogel patch application (schematic timeline, Fig. 7A). Erythema and edema were assessed at 0, 2, 24, 72, and 120 h using ISO 10993-10 (0 to 4 scale). Both PVA/PAM/LiCl/PDA and PVA/PAM/LiCl/PDA/LiH hydrogel groups, like the negative control, showed scores of 0 at all time points. In contrast, the positive control had sustained reactions (scores reaching 3 by 24 h, persisting to 120 h; Table S3), with representative images in Fig. 7B. As shown in Fig. 7C, hematoxylin and eosin (H&E) staining revealed distinct inflammatory cell infiltration patterns across treatment groups. Skin sections from the positive control group exhibited pronounced inflammatory pathology, characterized by robust eosinophil and mast cell influx. Quantitative evaluation showed drastically elevated cell densities in the positive control (eosinophils: 660 ± 173 cells/mm^2^; mast cells: 460 ± 104 cells/mm^2^), corresponding to ~11.0- and 3.8-fold increases relative to the negative control (60 ± 55 and 120 ± 76 cells/mm^2^, respectively; both *P* < 0.0001). In contrast, PVA/PAM/LiCl/PDA and PVA/PAM/LiCl/PDA/LiH hydrogel groups displayed histopathological profiles statistically indistinguishable from the negative control (*P* > 0.05), with inflammatory cell counts maintained at baseline levels (80 ± 87 and 100 ± 71 cells/mm^2^; 60 ± 55 and 120 ± 84 cells/mm^2^, respectively; Fig. 7D and E). Epidermal and dermal architectures remained intact in hydrogel-treated samples, with no pathological immune cell accumulation detected. In summary, these results indicated that the PVA/PAM/LiCl/PDA-based hydrogels, both with and without lidocaine, induced no detectable skin irritation or sensitization under the experimental conditions.

## Conclusion

We successfully prepared a novel biomass-composite PVA/PAM/LiCl/PDA/LiH hydrogel patch through a facile one-pot synthesis that rapidly crosslinked PVA, AM, and PDA. This hydrogel exhibited stretchability (534.22%), low modulus (0.044 kPa), tissue adhesion (1.82 kPa), high conductivity (3.90 S m^−1^), rapid self-healing ability, excellent biocompatibility, and antibacterial properties. The hydrogel patch was used to fabricate a sensor capable of finely sensing strain and body motion. With high sensitivity (GF = 5.31) and reliable long-term stability over 500 cycles, the sensor could quickly and accurately differentiate between several weak and strong human motions. Furthermore, we demonstrated that the patch, when integrated with deep learning algorithms, demonstrated high accuracy in assessing clinical conditions involving various types of pain, all without causing patient discomfort. Moreover, the patch provided a long-lasting, on-demand analgesic effect via photothermal activation on mice, as shown by PWT (>31% vs. Ctrl) and CPS (<10). PVA/PAM/LiCl/PDA-based hydrogels showed no detectable skin irritation or sensitization. We believe that this pioneering study will promote the application of portable wearable “smart band-aids” for pain diagnosis and treatment, and possesses vital innovativeness and potential for clinical application transformation. Notably, this study has some limitations. While the PVA/PAM/LiCl/PDA/LiH device distinguishes typical limited movements and pains, its utility for finer pain-field assessments requires further clinical validation. Additionally, the skin patch depends on external power and data acquisition hardware, which limits its overall wearability and portability. Future integration biomass-based hydrogel with portable hardware and wireless modules is envisioned for automated monitoring and assessment.

## Materials and Methods

Detailed information about the materials and methods used for this work are provided in the Supplementary Materials.

## Data Availability

All data supporting the findings of this study are available within the paper and its Supplementary Materials. In addition, the datasets generated or analyzed during this study are available from the corresponding authors on reasonable request.

## Supplementary Materials

Supplementary 1 Experimental Section Figs. S1 to S24 Tables S1 to S3 Movies S1 and S2
