# Supplementary material for: A Biomass-Inspired Hydrogel Patch for Intelligent Pain Monitoring and On-Demand Analgesia
Source: Research (Wash D C). 2026 Feb 11;9:1112. doi: 10.34133/research.1112 (PMC12891368; doi:10.34133/research.1112)
Supplement: Supplementary 1 — Experimental Section Figs. S1 to S24 Tables S1 to S3 Movies S1 and S2 [file research.1112.f1.zip › Supporting+Information-Research.docx]

**Supporting Information**

A Biomass-Inspired Hydrogel Patch for Intelligent Pain Monitoring and On-Demand Analgesia

Yibin Lin^1#^, Yan Wu^2#^, Haiting Fan^2#^, Yuling Wu^1#^, Hongcai Liang^3^, Wenjing Lin^1,4^*, Liangtian Lan^2^, Duoqu Chen^1^, Jiaxin Li^1^, Xia Feng^2^*, Shuai Zhao^3^*, Guobin Yi^1,4^*

1 School of Chemical Engineering and Light Industry, Guangdong University of Technology, Guangzhou, 510006, China

2 Department of Anesthesiology, First Affiliated Hospital, Sun Yat-sen University, Guangzhou, 510080, China

3 The Second Affiliated Hospital of Guangzhou University of Chinese Medicine, Guangzhou, 510120, China

4 Guangdong Provincial Laboratory of Chemistry and Fine Chemical Engineering Jieyang Center, Jieyang 515200, China

Experimental Section

**Materials**

Dopamine hydrochloride (DA, 98%), lidocaine hydrochloride (LiH, 99%), poly(vinyl alcohol) (PVA, alcoholysis degree: 87.0-89.0 mol%, viscosity: 20.0-30.0 mPa s), ammonium persulfate (APS, ＞99.0%), ammonium hydroxide solution (NH_3_·H_2_O, 25-28%) were purchased from Shanghai Macklin Biochemical Co., Ltd. Sodium hydrogen phosphate dodecahydrate (Na_2_HPO_4_⋅12H_2_O, 99%) and sodium phosphate monobasic dihydrate (NaH_2_PO_4_⋅2H_2_O, 99%) were purchased from Shanghai BiDe Pharmaceutical Technology Co., Ltd. N, N’-methylenebisacrylamide (MBA, 99%), acrylamide (AM, ≥99%), lithium chloride (LiCl, ≥99%) were purchased from Shanghai Aladdin Biochemical Technology Co., Ltd. Super-Enhanced Cell Counting Kit-8 was purchased from Dalian Meilun Biotechnology Co., Ltd. Penicillin-streptomycin and bovine serum albumin were purchased from Sigma-Aldrich (Shanghai) Trading Co.Ltd. Foetal bovine serum was purchased from Serana Europe GmbH. Dulbecco's Modified Eagle Medium (DMEM, Gibco) was purchased from Thermo Fisher Scientific Inc. The water used in all experiments was purified using a water purification system (Shanghai Zhiang BEST-S30) with resistivity higher than 18 MΩ cm^−1^.

**Synthesis of PDA nanoparticles**

Specifically, 1 mL of NH_3_·H_2_O and 140 mg APS was added to 35 mL of ethanol/water mixed solvents ($\text{V}_{\text{C}\text{H}_{\text{3}}\text{C}\text{H}_{\text{2}}\text{OH}}$/$\text{V}_{\text{H}_{\text{2}}\text{O}}$=2:7) while stirring, stirring vigorously for 0.5 h. Then, slowly add 10 mL of aqueous DA solution（22.5 mg mL^−1^） to the above solution. Subsequently, the PDA nanoparticle solution was obtained by stirring slowly at room temperature for 24 h. PDA nanoparticles were collected by high-speed centrifugation (10,000 rpm, 30 min), washed 3 times each with deionized water and absolute ethanol, and freeze-dried to obtain PDA nanoparticles. Finally, the prepared PDA nanoparticles were prepared into a 10 mg mL^−1^ aqueous solution of PDA and stored in the refrigerator (4 °C) for long-term use.

**Fabrication of PVA/PAM/LiCl/PDA hydrogel**

0.88 g of PVA was added to 13.5 mL of deionized water and stirred at 90 °C until the PVA molecules were completely dissolved. Then, a certain weight amount (0-1.25 mg mL^−1^) of PDA, AM (2.16 g), LiCl (0.75 g), MBA (0.0048 g), and APS (0.0640 g) was added to the PVA solution to obtain the reaction mixture, mixed evenly by sonication. The reaction mixture was filled into PTFE or silica mold, and the thickness of the final hydrogel was controlled by adjusting the amount of reaction mixture, and the PVA/PAM/LiCl/PDA hydrogel was obtained by heating at 80 °C for 50 min. The final products were denoted as PVA/PAM/LiCl/PDA_x_, where x (mg mL^−1^) represented the PDA concentration.

**Fabrication of PVA/PAM/LiCl/PDA/LiH hydrogel**

LiH (20 mg mL^−1^) was added to 0.5 mL of deionized water, and then 1.5 mL of PDA (10 mg mL^−1^) solution was added to above solution with stirring at room temperature for 12 h in the dark to obtain PDA-LiH solution. 0.88 g of PVA was added to 13.5 mL of deionized water and stirred at 90 °C until the PVA molecules were completely dissolved. Then, 0.75 g of LiCl, 2.16 g of AM, and PDA-LiH solution were added to the PVA solution. Finally, 0.0048 g MBA and 0.0640 g APS were added to the reaction mixture and kept at 80 °C for 50 min to obtain the PVA/PAM/LiCl/PDA/LiH hydrogel, which was stored at 4 °C for later use.

**Materials characterization**

The microstructure of the samples was characterized by scanning electron microscopy (SEM, TESCAN MIRA LMS, ​Czech) and transmission electron microscopy (TEM, JEM-F200, JEOL, Japan). The chemical composition and molecular structure of the samples were analyzed using a Thermo Scientific™ Nicolet™ iS50R advanced Fourier Transform Infrared Spectrometer (FTIR). For FTIR testing, samples were mixed with high-purity potassium bromide (KBr), pressed into pellets, and scanned 32 times at 25 °C to collect spectra from 400 to 4000 cm^−1^. Optical images were taken with an iPhone 12.

**Mechanical properties test**

The mechanical properties were evaluated using a universal testing machine (Inspekt Table Blue 5 kN, Hegewald & Peschke, Germany) with a 500 N load sensor at room temperature. The dimensions of the tensile test samples were 50 mm × 10 mm × 2 mm (length × width × thickness), and the tensile speed was 100 mm min^−1^. The compression tests were conducted on the same machine equipped with a 500 N load sensor and a speed of 5 mm min^−1^ at room temperature, using the samples with a height of 10 mm and a diameter of 20 mm. Elastic modulus was calculated from the linear slope of the elastic region between 0-20% strain in stress-strain curves. Toughness was calculated according to the area under the stress-strain curves. For cyclic tensile/compression loading-unloading tests, the hydrogel sample was repeatedly loaded to a certain strain and then unloaded at the same strain speed without any rest time. The dissipated energy can be obtained by measuring the area of the hysteresis return lines.

**Adhesive properties test**

The adhesive properties of the hydrogel were quantified through lap shear testing performed on a universal testing machine (CMT5105, Zhuhai, China) equipped with a 50 N load sensor. Test specimens measuring 40 mm in length, 20 mm in width, and 2 mm in thickness were utilized. Porcine skin, sized at 30 mm × 70 mm, served as the substrate. Adhesion strength was determined at a speed of 15 mm min^−1^ in an environment controlled to 30 °C and 60% RH.

**Molecular dynamics simulation**

Molecular models of PAM, PDA, PVA, LiCl, and proteins were constructed for molecular dynamics (MD) simulations using Materials Studio software. Five molecular models were developed to simulate the interfacial interactions between PAM/PVA/LiCl/PDA and proteins and to calculate the cohesion energy density in the PAM/PVA/LiCl/PDA system. Each model contains five fragments: PDA, PAM, PVA, H_2_O, and LiCl. In this paper, human skin was simulated using an all-atom model of the fibronectin fragment (1fbr.pdb) from the Protein Data Bank (PDB) website for type I collagen, which makes up >80% of the skin. To investigate the effect of PDA content on polymer properties, a polymer system consisting of PVA, PAM, LiCl, H_2_O, and five PDA molecules with different mass concentrations (0.25, 0.50, 0.75, 1, and 1.25 mg mL^−1^) was established. Separately, the polymer and protein layers were constructed, respectively. The interfacial adsorption model between PAM/PVA/LiCl/PDA and proteins was constructed by combining the protein layer with the above five molecular models.

MD simulations were conducted utilizing the condensed-phase optimized potential with atomic charges (COMPASS II) force field. The coulombic and van der Waals interactions within the system were calculated employing both Ewald summation and atom-based approaches. Under a constant temperature and volume (NVT) ensemble, the model system was subjected to an initial simulation at a temperature of 298 K, with a time step of 500 ps, to achieve an equilibrium state. Subsequently, an additional MD simulation was executed with a time step of 500 ps for data collection following the equilibration. Temperature regulation during the MD simulations was achieved by applying a Nosé-Hoover thermostat. Post-simulation analysis focused on the equilibrium configurations of interactions within various hydrogel-protein systems.

Cohesive Energy Density (CED) is the sum of the intermolecular energies per unit volume and is a quantitative measure of the energy of cohesion of a substance at equilibrium value, which depends on the interactions within the polymer. The following formula (1) can be calculated:

|  | $\text{CED=}\frac{\text{E}_{\text{cohesion}}}{\text{V}}$ | (1) |
| --- | --- | --- |

Where $\text{E}_{\text{cohesion}}$ and *V* denote the cohesion energy and volume, respectively, which $\text{E}_{\text{cohesion}}$ can be calculated according to the following formula (2):

| $\text{E}_{\text{cohesion}}\text{=}{\text{-}\text{E}}_{\text{intermolecular}}\text{=}\text{E}_{\text{intramolecular}}{\text{-}\text{E}}_{\text{total}}$ | (2) |
| --- | --- |

Where $\text{E}_{\text{intermolecular}}$, $\text{E}_{\text{intramolecular}}$, and $\text{E}_{\text{total}}$ refer to the intramolecular energy, the intramolecular energy between all molecules, and the total energy of the system, respectively ([Table S1](#TableS1)).

The interfacial adhesion energy (IAE) between hydrogel and protein can be calculated using the following formula (3):

| $\text{E}_{\text{adhesion}}\text{=}\text{E}_{\text{total}}\text{-}\text{E}_{\text{A}}{\text{-}\text{E}}_{\text{B}}$ | (3) |
| --- | --- |

Where $\text{E}_{\text{adhesion}}$, $\text{E}_{\text{total}}$, $\text{E}_{\text{A}}$, and $\text{E}_{\text{B}}$ denote the energy of interfacial interaction between hydrogel and protein, the total energy of the hydrogel-protein system, the energy of PAM/PVA/LiCl/PDA-H_2_O, and the energy of the protein layer, respectively (Table S1).

**Rheological and self-healing properties test**

Hydrogel samples were fabricated in circular discs with a diameter of 25 mm and a thickness of 2 mm. The rheological properties of these hydrogels were determined using a rheometer (Anton Paar Physica MCR 301, Graz, Austria). Three different rheological tests were performed: (1) Strain sweep measurement was conducted to obtain the linear viscoelastic region of the hydrogels at 37 °C with a constant frequency of 1 Hz. (2) Frequency sweep experiment was carried out at 37 °C, under a constant strain of 1%, spanning a frequency range from 0.1 Hz to 10 Hz. (3) Dynamic step-strain sweep test was performed to investigate the self-healing behavior of hydrogels by determining the storage modulus (G′) and loss modulus (G″) at 1 % and 2000 % oscillating strains. For the cut-healing experiments, hydrogel disks were cut in half and self-healed for 4 hours at 26 °C and 60% RH. For the light bulb test, the circuit consisted of a direct current power supply, a diode, a hydrogel, and some conductors to record the illuminance variation of the diode during the cutting and healing process of the hydrogel. For the conductive self-healing test, resistance changes during the cutting and healing cycles were recorded using a digital multimeter (Keithley 2000, Keithley Instruments, Inc., USA). For the sensing self-healing test, relative resistance variations of the original and self-healing (at 25 °C for 48 h) hydrogels were determined by combining a universal testing machine (CMT5105, Zhuhai, China) and a digital multimeter (Keithley 2000, Keithley Instruments, Inc., USA). Relative resistance changes were calculated by the following formula (4):

|  | ${\text{Δ}\text{R}}/{\text{R}_{\text{0}}}\text{=}\frac{\text{R}\text{-}\text{R}_{\text{0}}}{\text{R}_{\text{0}}}$ | (4) |
| --- | --- | --- |

In which *R*(Ω) and *R_0_*(Ω) were the real-time resistance and initial resistance of the hydrogel, respectively.

**Conductive and sensing properties test**

Electrical characterization was conducted utilizing an electrochemical workstation (CHI660D, CH Instruments, Inc., USA). The electrochemical impedance spectroscopy (EIS) of the hydrogel samples (50 mm × 10 mm × 2 mm) was assessed within a frequency range of 1 Hz to 10^6^ Hz, applying a voltage of 5 mV to determine the resistance (*R*). The conductivity (S m^−1^) of the hydrogels was subsequently derived using formula (5):

|  | $\text{Conductivity=}\frac{\text{L}}{\text{S}\text{×}\text{R}}$ | (5) |
| --- | --- | --- |

Where *L*(m), *S*(m^2^), and *R*(Ω) represented the distance between the electrode sheets, the cross-sectional area, and the resistance of the hydrogels, respectively.

Besides, the circuit consisted of a direct current power supply, a diode, a hydrogel, and some conductors to record the illuminance variation of the diode during the stretching and releasing processes of the hydrogel.

The relative resistance variations (Δ*R*/*R_0_*) of the hydrogels subjected to different strains and frequencies were quantified utilizing a universal testing machine (CMT5105, Zhuhai, China) in conjunction with a precision digital multimeter (Keithley 2000, Keithley Instruments, Inc., USA).

**Anti-freezing properties test**

The hydrogel samples were subjected to cyclic tensile and sensing stability tests by placing them in a −10 °C temperature controller attached to a universal testing machine (Inspekt Table Blue 5 kN, Hegewald & Peschke, Germany) for 1 h.

**Detection of joint diseases**

First, a hydrogel patch was immobilized to the specific detection area of the subject. Subsequently, participants were required to perform a series of predefined standardized movements, which were repeated several times, and the electrical changes resulting from the movements were captured and recorded in real-time. During the execution of the movement sequence, when the subjects reported feeling significant discomfort or restriction of movement, the current movement should be stopped immediately without forcing the entire predetermined movement sequence. That point was considered the termination point of the current movement cycle for that individual. At this point, the subject should return to the starting position. The informed written consent from all participants was obtained prior to the research. The study did not involve blood collection to humans or invasive procedures, all experiments were limited to skin surface contact tests.

**Design of deep learning framework based on Convolutional Neural Networks**

The core of the proposed method for quantitative pain assessment is a multi-layer Convolutional Neural Network (CNN) designed to extract features and automatically identify input signals. The network includes several specialized modules, such as convolutional layers, C2f modules, Spatial Pyramid Pooling with Fusion (SPPF), Path Aggregation Network (PAN), Feature Pyramid Network (FPN), fully connected layers, activation layers, pooling layers, normalization layers, upsampling layers, and output layers. Additionally, a densely connected classifier with dropout regularization is appended to network’s end to enhance generalization and prevent overfitting. The backbone of the model is based on the YOLOv8 framework, which consists of three main components: Backbone, Neck, and Head. The Backbone is responsible for feature extraction, the Neck enhances the extracted features through mechanisms like PAN and FPN, and the Head performs the final classification or regression tasks. Waveform data were collected using a digital multimeter (Keithley 2000), focusing on shoulder and lumbar movements as target categories. To ensure the robustness and generalization of the model, the original dataset was divided into training, validation, and test sets in a ratio of 1:1:1. The training set was used for parameter learning and optimization, the validation set for monitoring the model's performance on unseen data and tuning hyperparameters, and the test set for the final evaluation of the model's performance after training. During the model training process, classification accuracy, rate, and loss functions were continuously monitored to assess the model's learning progress and effectiveness.

**Photothermal properties test**

To evaluate the photothermal performance of PDA NPs and their hydrogels, an 808 nm NIR laser was utilized. Initially, a 1 mg mL^−1^ solution of PDA NPs was irradiated at a power density of 0.30 W cm^−2^. Subsequently, hydrogel samples containing varying concentrations of PDA NPs (0 to 1.25 mg mL^−1^) with dimensions of 30 mm × 30 mm × 2 mm were prepared and tested under a fixed power density of 0.30 W cm^−2^ to assess their photothermal properties. In addition, hydrogel samples with dimensions of 30 mm × 30 mm × 1.8 mm were fabricated and irradiated using different power densities ranging from 0.1 to 0.30 W cm^−2^ while maintaining a constant PDA NPs concentration. Throughout the experiments, temperature changes were recorded using an online infrared thermography system, the MAG14 (Shanghai Juge Technology Co., Ltd.), and the photothermal stability of PVA/PAM/PDA/LiCl hydrogels during heating and cooling cycles was investigated.The photothermal conversion efficiency is calculated using formula (6), (7), and (8):

| $\eta=\frac{hS\left( T_{max}-T_{s} \right)-Q_{0}}{I(1-{10}^{(-A)})}$ | (6) |
| --- | --- |
| $hS=\frac{mc}{\tau}$ | (7) |
| $t=-\tau ln(\theta)\frac{T-T_{s}}{T_{max}-T_{s}}$ | (8) |

*η* is the photothermal conversion efficiency, *h* is the heat transfer coefficient, *S* is the surface area of the container, *T_max_* is the equilibrium temperature, *T_s_* is the ambient temperature, and *T* is the temperature of the sample at a certain moment during the cooling process. *Q_0_* refers to the heat dissipated by the light absorbed by the sample cell, which can be neglected. *I* is the laser power density (0.3 W/cm^2^), *A* is the absorbance of the hydrogel sample at 808 nm, *m* is the mass of the solvent, and *c* is the specific heat capacity of the solvent (4.2 J/(g·°C)). τ is the time constant of the sample, which can be derived via linear regression of the cooling time (*t*) and −ln(*θ*) of the hydrogel.

**In vitro antibacterial properties test**

Hydrogel samples with a thickness of 2 mm and a diameter of 7 mm were prepared, underwent UV sterilization, and were placed in a 24-well plate. A bacterial suspension (10 μL, 10^7^ CFU mL^−1^) was applied to the surface of each sample, and 1 mL of phosphate buffered saline (PBS) was added to each well. The samples were divided into two groups: the NIR-irradiated group and the non-irradiated group. For the NIR-irradiated group, the samples were subjected to NIR light irradiation at an intensity of 0.30 W cm^−2^ for 10 minutes, while the non-irradiated group did not undergo NIR light irradiation. Both groups were then incubated at 37 °C for 1 hour. After incubation, the bacterial suspension from each well was diluted 10^2^ times, and 30 μL of the diluted suspension was evenly spread on agar plates. The plates were incubated at 37 °C for 24 h, and the number of bacterial colonies was counted to obtain the data.

**In vitro drug release test**

The transdermal diffusion efficiency of the PVA/PAM/PDA/LiCl/LiH hydrogel was assessed using an enhanced Franz diffusion cell method[[1]](#Ref1). The hydrogel samples were placed on a 10% gelatin skin model in the donor compartment. The receptor compartment contained 8 mL of PBS (pH 7.4) at 32 °C, stirred at 300 rpm. Samples (200 μL) were collected at intervals (0 h, 0.5 h, 1 h, 2 h, 4 h, 8 h) for UV-Vis analysis, with fresh PBS replenished after each withdrawal. Similarly, the photothermal drug release behavior of the PVA/PAM/PDA/LiCl/LiH hydrogel was investigated by exposing the hydrogels to NIR light (0.30 W cm−²) perpendicular to their surface. At set time points, 200 μL of PBS solution was collected and replaced with fresh PBS.

**In vitro cytotoxicity assay**

10 mg of PVA/PAM/LiCl/PDA and PVA/PAM/LiCl/PDA/LiH were separately dissolved in 10 mL of culture medium, soaked for 24 h and then filtered to obtain hydrogel extract (1000 mg L−1). CCK-8 assay was used to analyze the cytotoxicity of PVA/PAM/LiCl/PDA and PVA/PAM/LiCl/PDA/LiH on mouse macrophage (Raw 264.7) cells and human stem fibroblast (HSF) cells. Specifically, Raw 264.7 and HSF cells were cultured in 96-well plates at a density of 0.5 × 104 cells well−1 and supplemented with 100 μL of cell culture medium (1% penicillin-streptomycin solution, 10% fetal bovine serum, and 89% DMEM). The 96-well plate was placed in a constant temperature and humidity incubator (37 °C, 5% CO2) for 24 h. After three washes with PBS, 100 μL of different concentrations of PVA/PAM/LiCl/PDA and PVA/PAM/LiCl/PDA/LiH extract were added to each well. After 24 h of cell culture, 10 μL of CCK8 solution was added to each well and incubated for 2 h. Finally, the absorbance of each well was measured at a wavelength of 450 nm using a UV spectrophotometer.

**Cell morphology analysis**

Actin-DAPI and Tubulin-DAPI staining was used to visualize the change in cell morphology. After 24 h treatment of PVA/PAM/LiCl/PDA and PVA/PAM/LiCl/PDA/LiH, HSF or Raw 264.7 cells were fixed in 4% paraformaldehyde for 30 min and permeabilized using 0.3% Triton X-100 solution for 15 min. Then, samples were blocked with 5% bovine serum albumin (BSA) for 30 min at room temperature, washed with PBS, and incubated with primary antibodies (Actin 1:200, Tubulin 1:200, Affinity) overnight at 4 °C. After three washes with PBS, cells were incubated with secondary antibodies (Goat anti-Rabbit Alexa Fluor 555 1:200, Goat anti-Rabbit Alexa Fluor 488 1:200) for 1 h at 37 °C. Cell nuclei were stained with DAPI for 10 min. After staining, cells were imaged by a fluorescence microscope (Zeiss/Axio Observer7).

**Ethics declarations and grouping**

Male BALB/c mice (15-22 g) were provided by the Institute of Experimental Animals of Sun Yat-sen University. The mice were maintained in a specific pathogen-free (SPF) environment with a temperature range of 22 to 25 °C, humidity levels between 40% and 70%, and a 12-hour light/dark cycle. Food and water were available ad libitum. All experimental procedures were approved by the Animal Care and Use Committee of Sun Yat-sen University (Approval No.: 2024000150) and conducted strictly in accordance with the guidelines of the National Institutes of Health’s Guide for the Care and Use of Laboratory Animals. The mice were divided into four groups: Ctrl, PVA/PAM/LiCl/PDA, PVA/PAM/LiCl/PDA/LiH, and PVA/PAM/LiCl/PDA/LiH+NIR.

**Incision pain model**

To assess the analgesic efficacy of LiH, we developed a postoperative incision pain model[[2]](#Ref2). Mice were anesthetized with an intraperitoneal injection of sodium pentobarbital (50 mg kg−1). Using a surgical blade, a 0.5 cm longitudinal incision was made from the proximal edge of the heel and extended toward the toes through the skin and fascia of the plantar aspect of the foot. The skin was sutured with surgical sutures, rinsed with normal saline, disinfected with 75% alcohol, and the mice were placed in a single cage for recovery.

**Pain withdrawal threshold test (PWT)**

The PWT of mice, assessed using von Frey filaments, is a standard behavioral test to quantify mechanical allodynia. The “up-down” method was employed, applying stimuli to the lateral plantar surface of the hind paw with filaments ranging from 0.40 g to 2.00 g (NC12775-99, Aesthesio, USA) to establish the 50% response threshold, defined as the minimum force eliciting at least five positive responses [[3]](#Ref3). Each filament was applied for 6-8 s, with a 10-second interval between applications to allow recovery from the previous stimulus. The testing was conducted using a blinded protocol, and all mice survived throughout the experiments. The PWT of mice was finished at 0 h, 2 h, 1 day, 2 day, 3 day and 5 day after surgery. Hydrogel patches were replaced once daily, and testing was conducted 2 h after patch application. For the NIR group, mice were exposed to NIR irradiation (0.3 W cm−2 for 10 min) prior to each assessment.

**Cumulative pain score (CPS)**

The CPS of mice was utilized to further assess pain behavior[[4]](#Ref4). The CPS of each group was evaluated at different time points depending on the position in which the foot was found during the scoring period, as follows: if the wound turned white or deformed when the paw touched the mesh, the mouse was considered to have no pain, and 0 score was recorded. If the paw did not touch the grid at all, it is considered that the mouse had a strong spontaneous pain and could not touch the ground, and 2 points were recorded. The remaining cases were recorded as 1 point. Scores were recorded every 5 minutes for 1 h, and CPS was obtained as the sum of 12 scores. Spontaneous pain thresholds were measured at 0 day, 2 h, 1 day, 2 day, 3 day, and 5 day.

**Skin contact safety assessment of hydrogels**

A skin irritation and sensitization study was performed using male BALB/c mice (8–10 weeks old, 20–25 g; n=5 per group). After dorsal hair removal, test materials were applied under occlusion with 7-mm patches. The treatments included filter paper moistened with saline as a negative control, 10% SDS-saturated filter paper as a positive irritant control, a lidocaine-free PVA/PAM/LiCl/PDA hydrogel, and a lidocaine-loaded PVA/PAM/LiCl/PDA/LiH hydrogel. Skin reactions were evaluated at 0, 2, 24, 72, and 120 hours post-application. Erythema and edema were each scored on a 0–4 scale according to the standardized criteria described in ISO 10993-10:2010 tests for irritation and skin sensitization. All test sites were photographed for documentation (iPhone 15).

**Histological and mast cell staining**

Upon completion of the 120‑hour observation period, the mice were terminally perfused with 4% paraformaldehyde for fixation. Full-thickness circular biopsies (10 mm diameter) centered on the hydrogel patches were then harvested from the dorsal skin of each mouse. Tissues were post-fixed in 4% paraformaldehyde for 24 h, followed by dehydration, paraffin embedding, and sectioning at 4 µm with a microtome (PM‑24, Servicebio, China). Sections were mounted on glass slides (G6012‑1, Servicebio) for subsequent staining. For hematoxylin and eosin (H&E) staining, sections were deparaffinized in xylene, rehydrated through graded ethanol, stained with hematoxylin and eosin, dehydrated, cleared, and coverslipped with neutral balsam.For mast cell detection, sections were stained with toluidine blue (G1032, Servicebio) for 2–5 minutes, briefly differentiated in 0.1% acetic acid, washed, air‑dried, and mounted. Mast cells were quantified in three randomly selected microscopic fields per section at 400× magnification using a light microscope (SWe‑CX63, Servicebio).

**Statistical analysis**

All experiments were carried out at least three times unless otherwise noted. The data were analyzed using Origin, followed by a Student’s t-test, one-way analysis of variance and two-way analysis of variance. **P* < 0.05 was considered statistically significant. ***P* < 0.01 , ****P* < 0.001 and *****P* < 0.0001were considered highly significant.

**
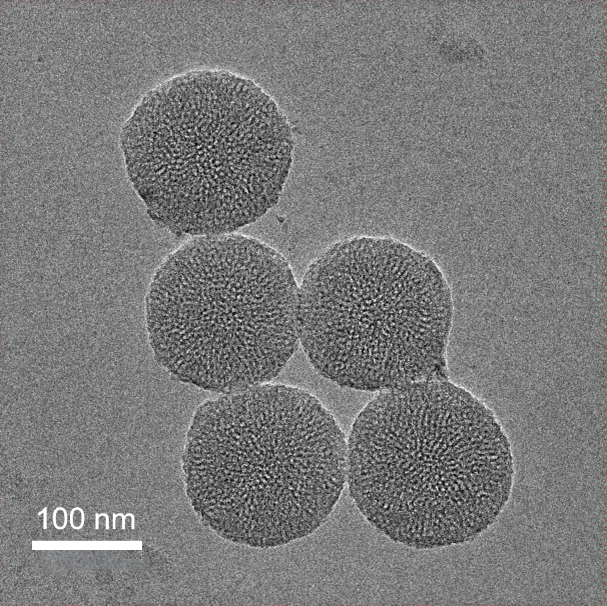
**

**Fig. S1.** TEM image of PDA nanoparticles.





**Fig. S2.** Photothermal effects of PDA.





**Fig. S3.** Tensile modulus of PVA/PAM/LiCl/PDA hydrogel with different PDA NPs contents.





**Fig. S4.** Compression modulus of PVA/PAM/LiCl/PDA hydrogels with different PDA NPs contents.

**
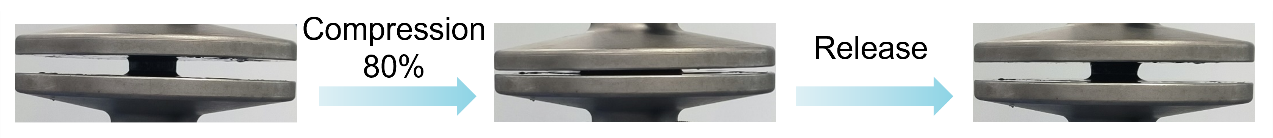
**

**Fig. S5.** Optical images of the PVA/PAM/LiCl/PDA_0.75_ hydrogel with compression state.





**Fig. S6.** The loading-unloading cycle of PVA/PAM/LiCl/PDA_0.75_ hydrogel at 10-50% tensile strains.





**Fig. S7.** The loading-unloading cycle of PVA/PAM/LiCl/PDA_0.75_ hydrogel at 100-400% tensile strains.





**Fig. S8.** The loading-unloading cycle of PVA/PAM/LiCl/PDA_0.75_ hydrogel at 10-65% compression strains.





**Fig. S9.** The dissipated energy and peak stress of PVA/PAM/LiCl/PDA_0.75_ hydrogel at 10-65% compression strains.


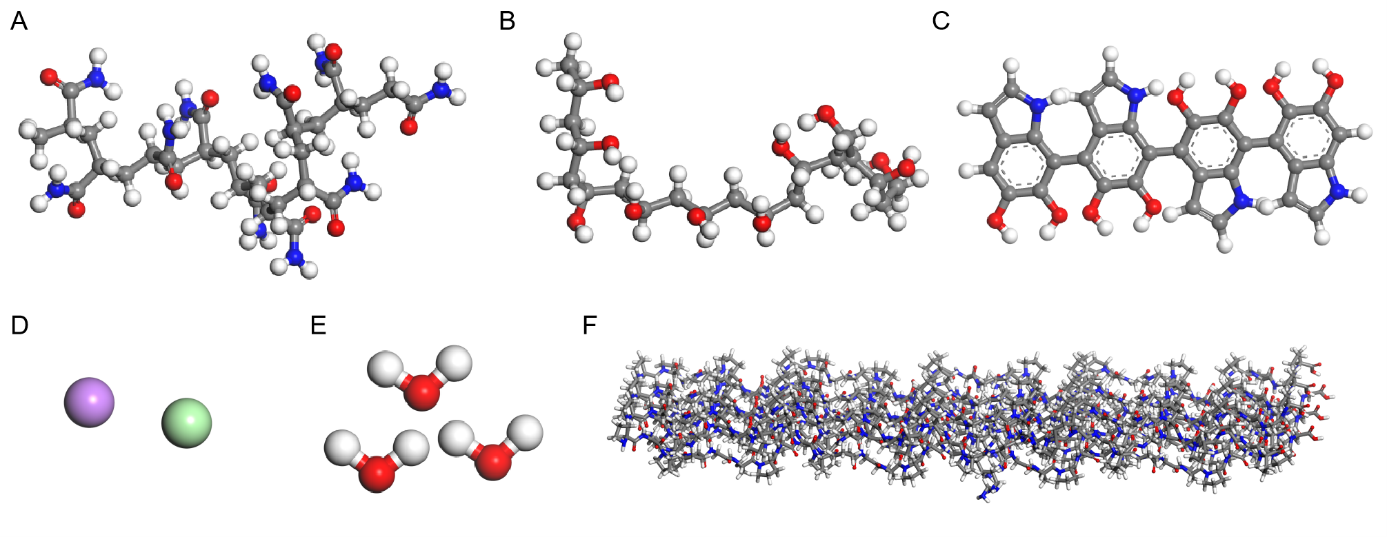


**Fig. S10.** MD simulation model. A) PAM. B) PVA. C) PDA. D) LiCl. E) H_2_O. F) Protein.

**
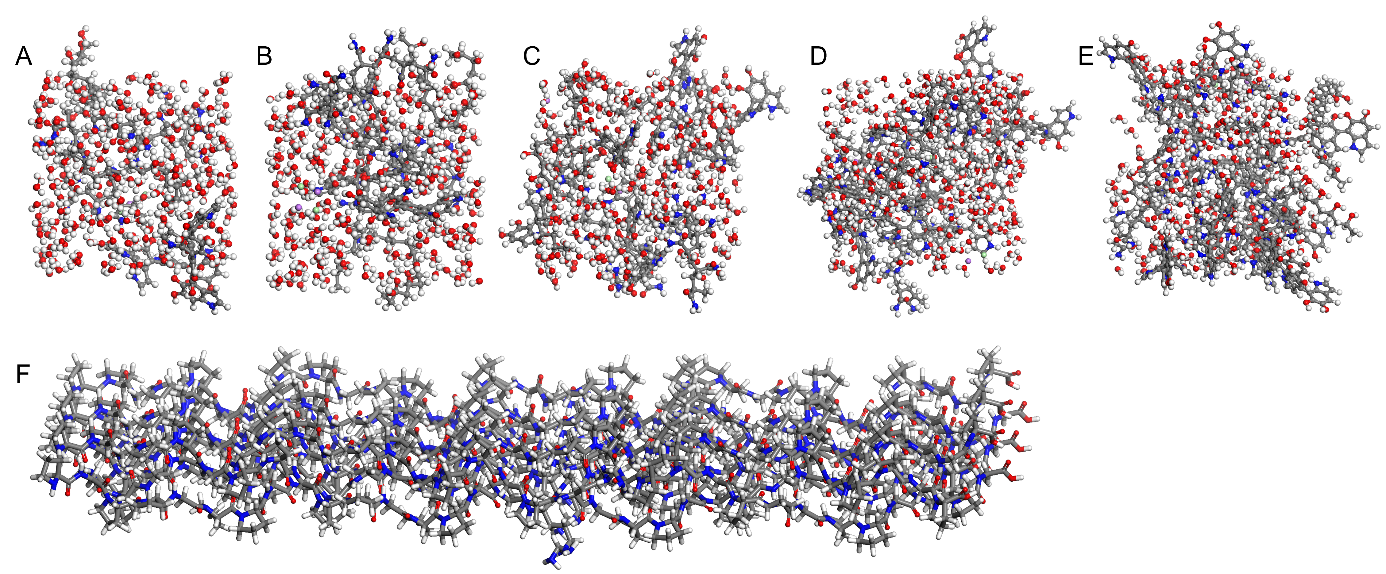
**

**Fig. S11.** Modeling of interfacial adsorption between a polymer layer composed of different PDA contents and a protein layer composition. A) PAM/PVA/LiCl/PDA_0.25_. B) PAM/PVA/LiCl/PDA_0.50_. C) PAM/PVA/LiCl/PDA_0.75_. D) PAM/PVA/LiCl/PDA_1.00_. E) PAM/PVA/LiCl/PDA_1.25_. F) Protein.


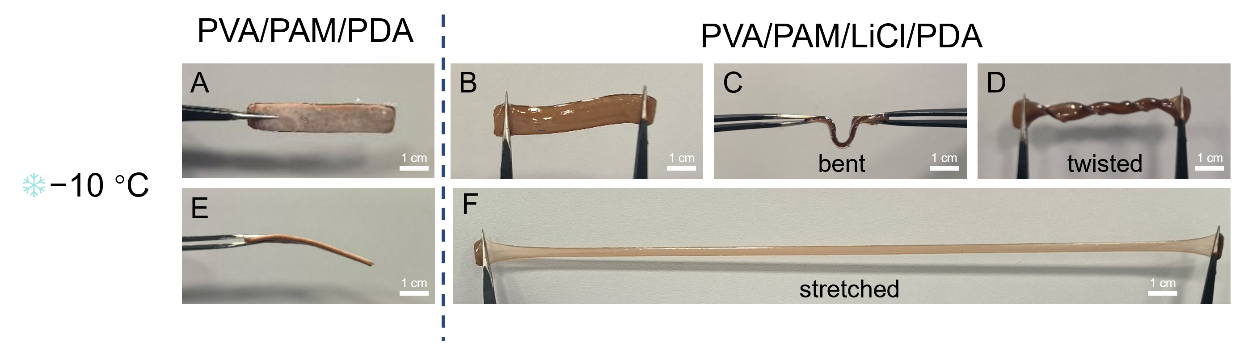


**Fig. S12.** Comparison of PVA/PAM/PDA and PVA/PAM/LiCl/PDA hydrogels after storing at −10 °C for 1 h. Optical image of PVA/PAM/PDA hydrogel with A) frozen and E) whitened state. Optical images of PVA/PAM/LiCl/PDA hydrogel with B) original, C) bent, D) twisted and F) stretched state.





**Fig. S13.** Loading-unloading curves of PVA/PAM/LiCl/PDA_0.75_ hydrogel at 50% strain for 50th cycles (−10 °C).





**Fig. S14.** Conductivity of the PVA/PAM/LiCl and PVA/PAM/LiCl/PDA hydrogels.


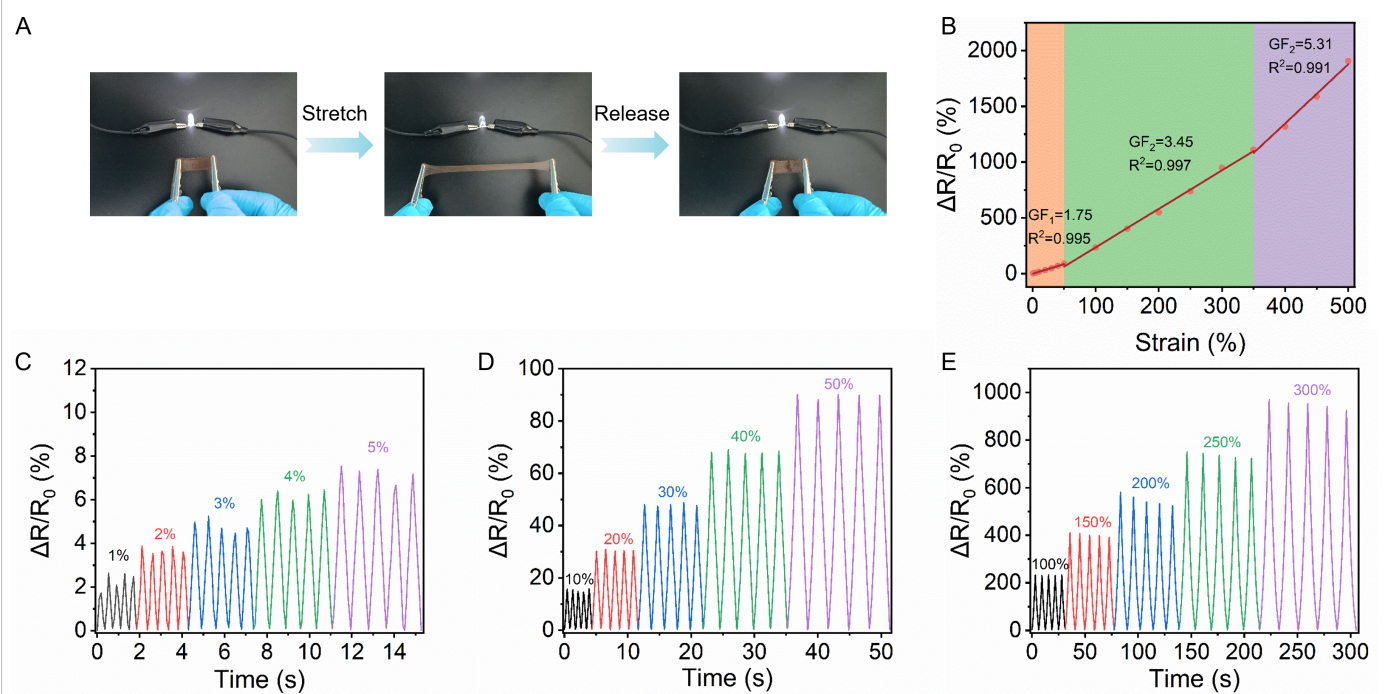


**Fig. S15.** A) Illuminance variation of the LED bulb during the stretch loading and unloading process of PVA/PAM/LiCl/PDA_0.75_ hydrogel. B) Relative resistance variation-strain curve of PVA/PAM/LiCl/PDA_0.75_ hydrogel. Relative resistance variation of PVA/PAM/LiCl/PDA_0.75_ hydrogel with C) 1–5% strains, D) 10–50% strains, E) 100–300% strains.


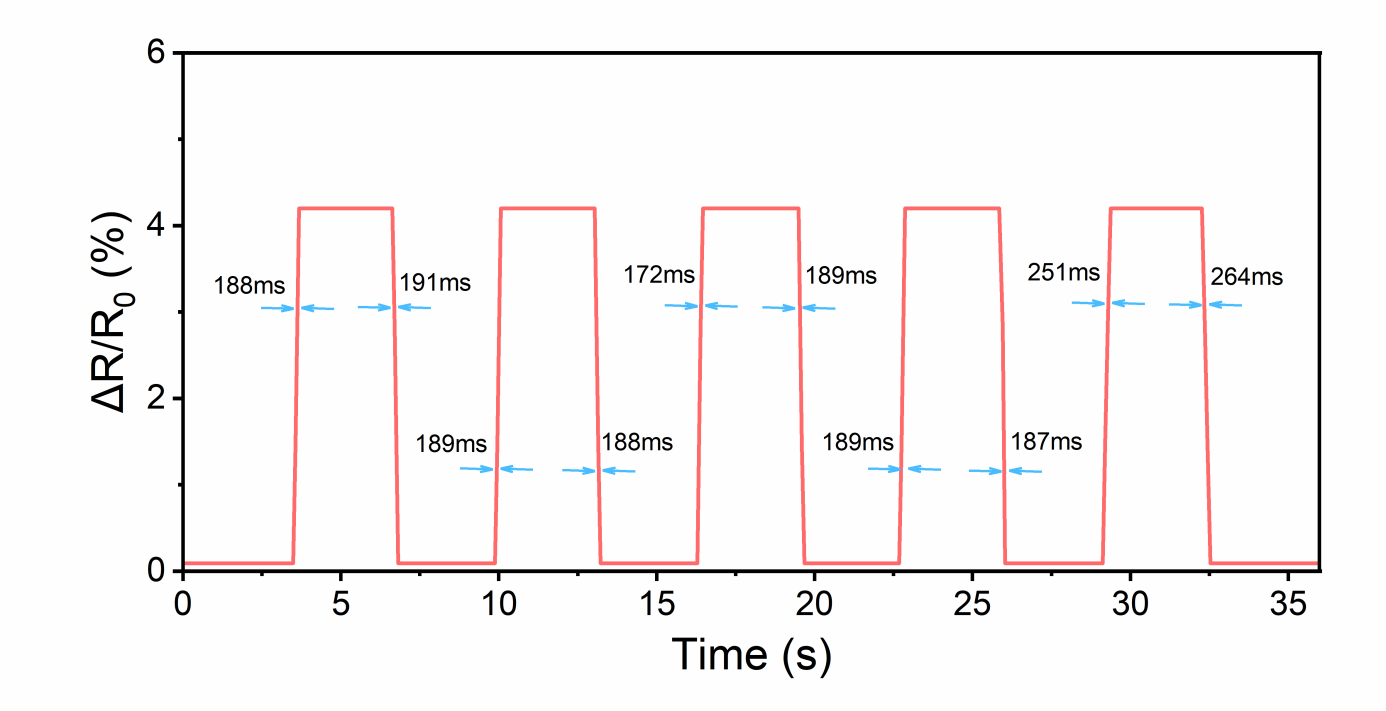


**Fig. S16.** The response time of the PVA/PAM/LiCl/PDA_0.75_ hydrogel during five loading-unloading cycles.


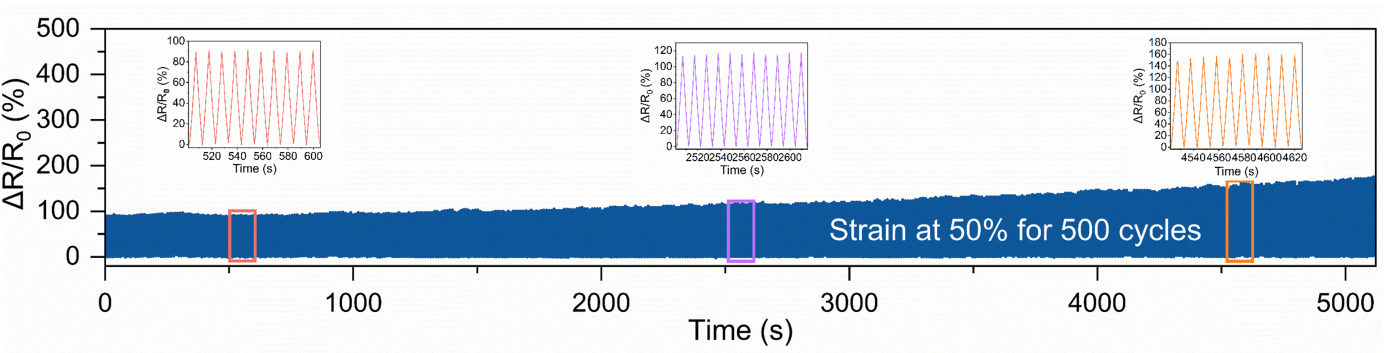


**Fig. S17.** Sensing stability test of PVA/PAM/LiCl/PDA_0.75_ hydrogel strain sensor at a strain of 50% for 500 tensile cycles.


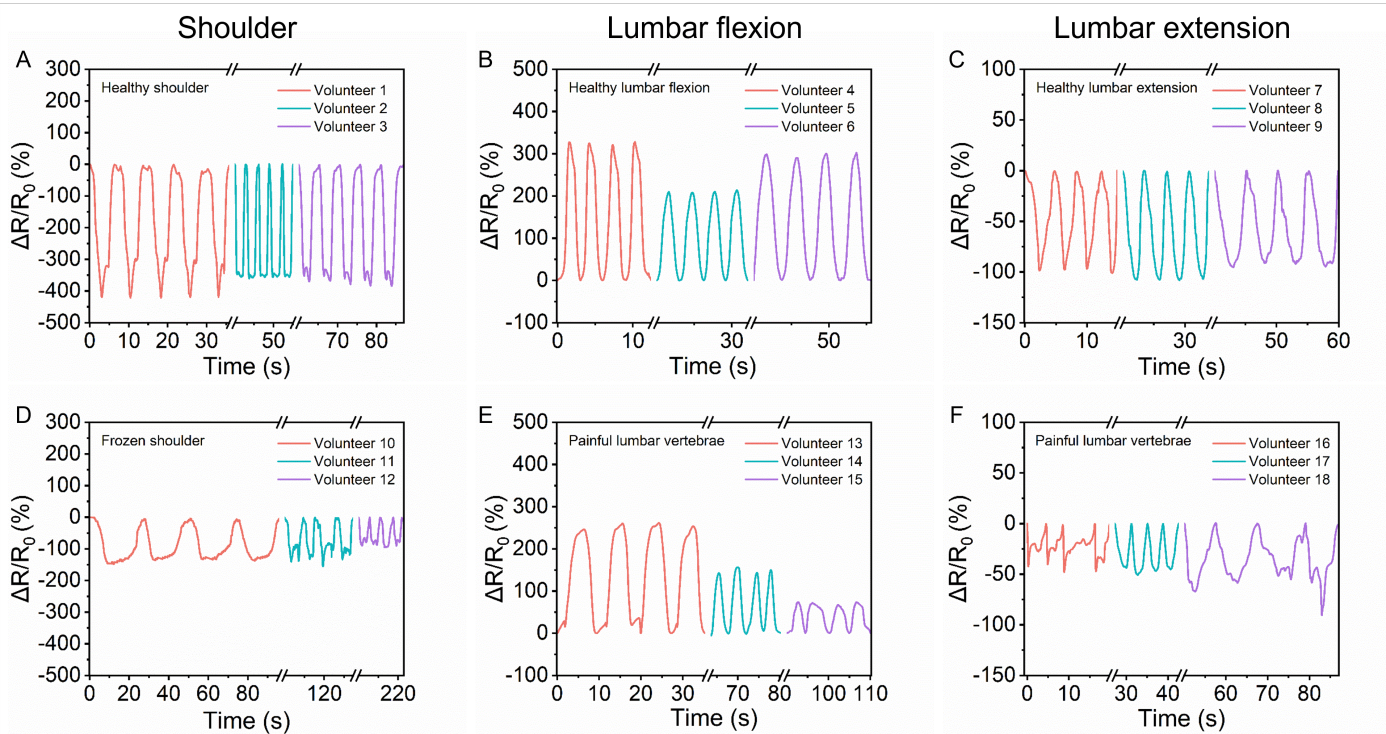


**Fig. S18.** The relative resistance variations monitored in real-time during A, D) shoulder joint movements, B, E) lumbar flexion, and C, F) lumbar extension in healthy and pain volunteers.


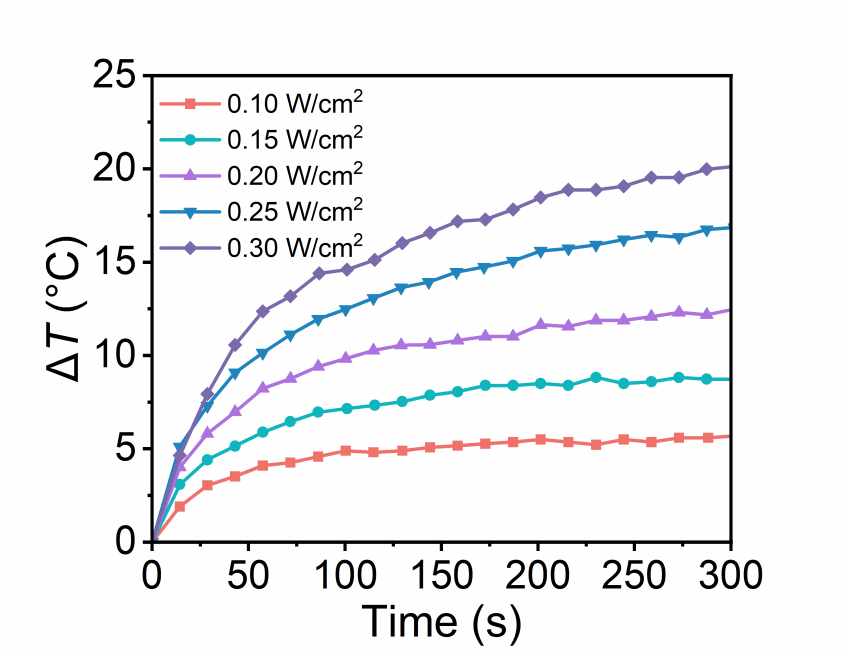


**Fig. S19.** Photothermal temperature rise curves under 808 nm NIR laser irradiation with different laser power densities.


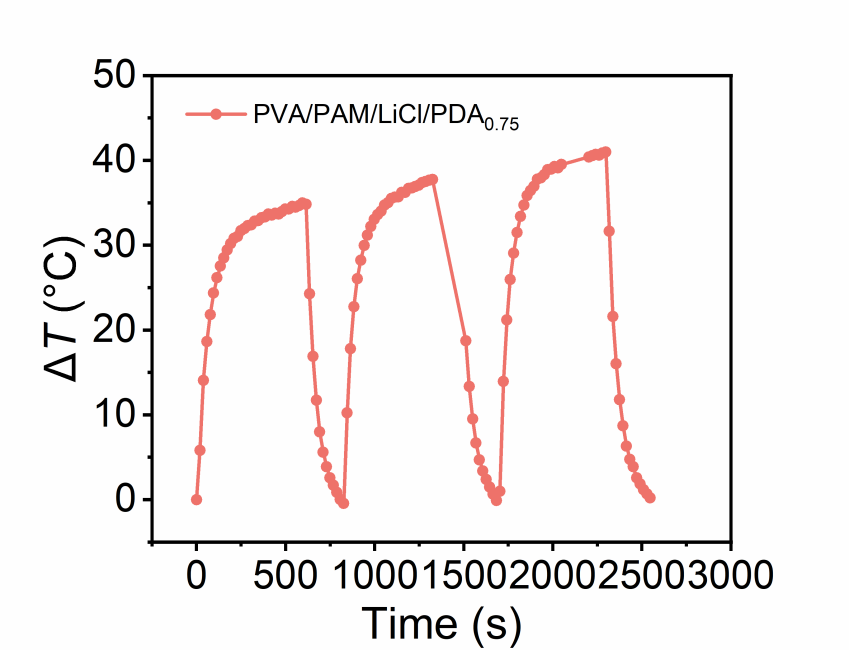


**Fig. S20.** Photothermal stability under repeated laser on-off of 808 nm NIR irradiation at a power density of 0.30 W cm^−2^.


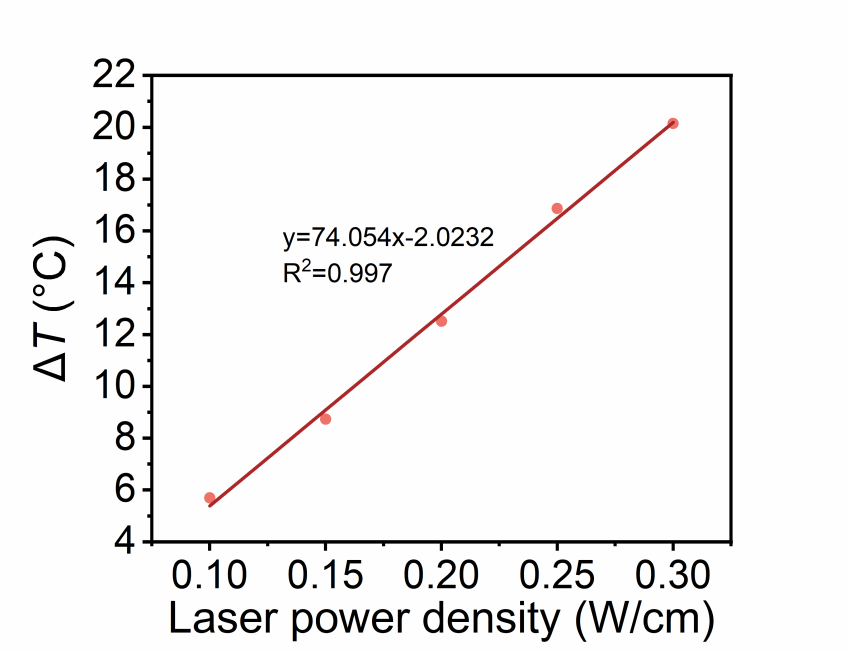


**Fig. S21.** Temperature change curves under 808 nm NIR laser irradiation at different laser power densities for 5 min.


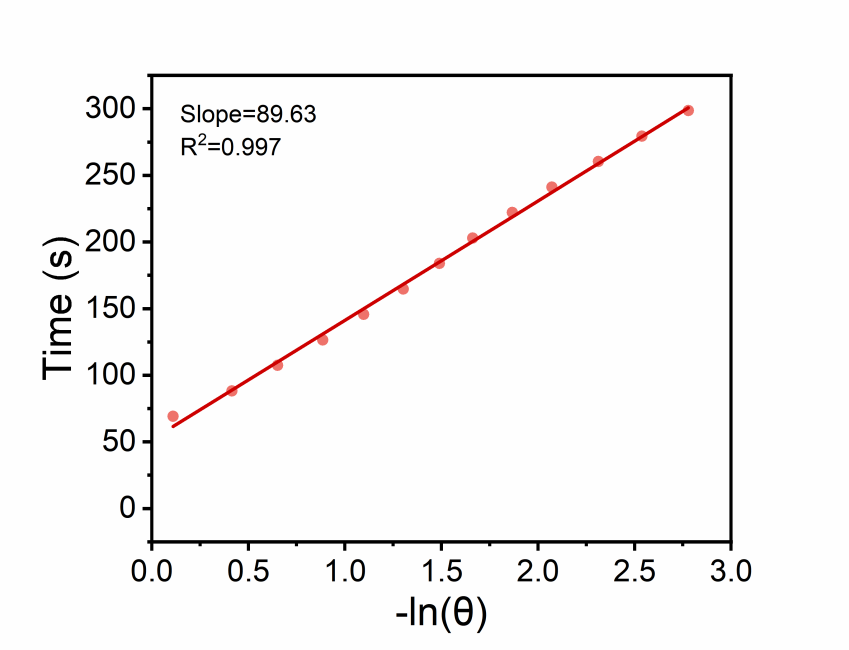


Fig.S22. The linear fitting of the temperature cooling time of PVA/PAM/LiCl/PDA_0.75_ hydrogel.


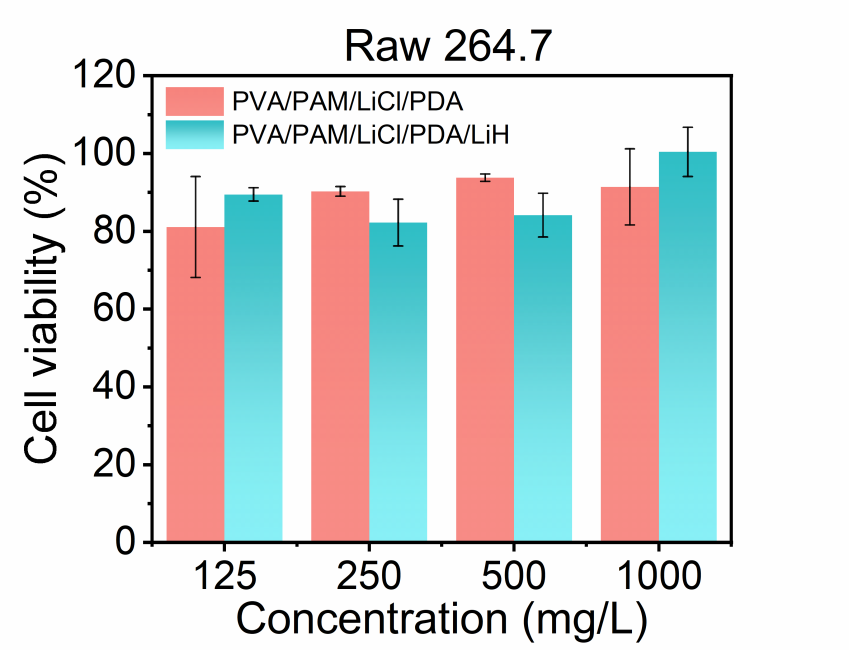


**Fig.23.** Relative viability of Raw 264.7 cells incubated with extracts of various concentrations of the PVA/PAM/LiCl/PDA and PVA/PAM/LiCl/PDA/LiH hydrogels after 24 h.


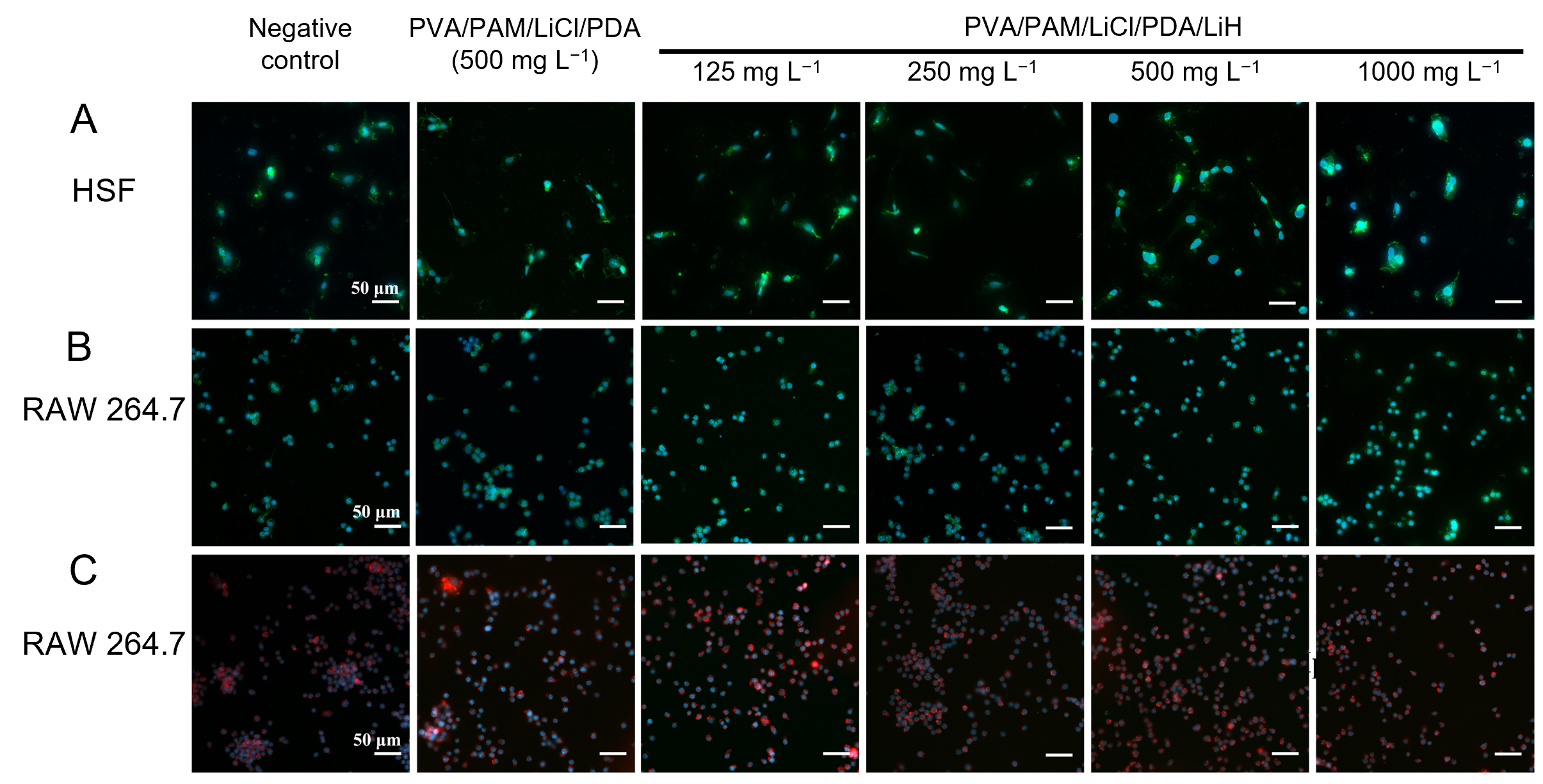


**Fig.24.** The morphologies of A) HSF cells or B) Raw 264.7 cells treated with PVA/PAM/LiCl/PDA and PVA/PAM/LiCl/PDA/LiH hydrogels stained with Tubulin-DAPI. C) The morphologies of Raw 264.7 cells treated with hydrogels stained with Actin-DAPI.

**Table S1.** The detailed energy during simulation calculations.

|  | **total**  **[kcal mol^−1^]** | **Protein**  **[kcal mol^−1^]** | **PAM/PVA/LiCl/PDA**  **[kcal mol^−1^]** | **IAE**  **[kcal mol^−1^]** | **CED**  **[GJ m^−3^]** |
| --- | --- | --- | --- | --- | --- |
| PAM/PVA/LiCl/PDA_0.25_ | −130356.416 | −128064.900 | −2213.280 | −78.236 | 0.281 |
| PAM/PVA/LiCl/PDA_0.50_ | −135798.359 | −128064.900 | −7561.628 | −171.831 | 0.314 |
| PAM/PVA/LiCl/PDA_0.75_ | −132522.123 | −128064.900 | −4007.299 | −449.924 | 0.362 |
| PAM/PVA/LiCl/PDA_1_ | −130534.194 | −128064.900 | −1925.540 | −543.754 | 0.413 |
| PAM/PVA/LiCl/PDA_1.25_ | −133828.497 | −128064.900 | −5238.536 | −525.061 | 0.919 |

**Table S2.** The conductivity of PVA/PAM/LiCl/PDA hydrogel was compared with the reported ionic conductive hydrogels.

|  | Conductive medium | Modulus [kPa] | Conductivity [S m^−1^] | Gauge factor | Self-healing ability | Biocompatibility | Antibacterial  effect | Ref. |
| --- | --- | --- | --- | --- | --- | --- | --- | --- |
| P(HPA-*co*-AM)/HPC/LiCl | Li^+^, Cl^-^  (1.27 wt%) | 38 | 1.0 | 1.11 | no | yes | no | [[5]](#Ref5) |
| Glu/P(HEA-*co*-AA)-Fe/LiCl | Li^+^, Cl^-^  (soak, 6 mol L^−1^) | 230 | 0.47 | – | yes | no | no | [[6]](#Ref6) |
| PAM-TSASN-LiCl | Li^+^, Cl^-^  (0.67 wt%) | – | 0.2 | 4.50 | no | no | no | [[7]](#Ref7) |
| P(AM-co-AA)/Trehalose/LiCl | Li^+^, Cl^-^  (4.36 wt%) | 14.89 | 4.13 | 3.59 | no | no | no | [[8]](#Ref8) |
| ASCL | Li^+^, Cl^-^  (7.21 wt%) | – | 0.27 | 1.07 | yes | no | no | [[9]](#Ref9) |
| β-CD-g-（pAAm/pAETAc） | 2-(acryloyloxy)ethyltrimethylammonium chloride | 14.1 ± 3.3 | 0.28 | 3.74 | yes | yes | no | [[10]](#Ref10) |
| SPE_2_NC_2.5_ | 2-(Methacryloyloxy) ethyl] dimethyl-(3-sulfopropyl) ammonium hydroxide | 62.2 | 0.015 | 0.8 | no | yes | no | [[11]](#Ref11) |
| PEDOT：PSS-PVA | PEDOT: PSS | – | – | 4.07 | no | no | no | [[12]](#Ref12) |
| A_2_LP-NC | (Me3N)4-TPE-4PF6 | – | 0.268 | 10.90 | no | yes | no | [[13]](#Ref13) |
| PAM-HBIMCP-Zn | Zn^2+^, CF_3_SO_3_^−^  (2.80 wt%) | 7.60 | 3.82 | 2.89 | no | no | no | [[14]](#Ref14) |
| **PVA/PAM/LiCl/PDA_0.75_** | **Li^+^, Cl^-^**  **(3.97 wt%)** | **0.044** | **3.90** | **5.31** | **yes** | **yes** | **yes** | **This work** |

**Table S3.** Results of erythema and edema values for each experimental group at different time points in the skin irritation test.

| Time point | Group | Erythema value | Oedema value |
| --- | --- | --- | --- |
| 0 h | Positive control | 0 | 0 |
|  | PVA/PAM/LiCl/PDA/LiH | 0 | 0 |
|  | PVA/PAM/LiCl/PDA | 0 | 0 |
|  | Negative control | 0 | 0 |
| 2 h | Positive control | 0 | 0 |
|  | PVA/PAM/LiCl/PDA/LiH | 0 | 0 |
|  | PVA/PAM/LiCl/PDA | 0 | 0 |
|  | Negative control | 0 | 0 |
| 24 h | Positive control | 3 | 2 |
|  | PVA/PAM/LiCl/PDA/LiH | 0 | 0 |
|  | PVA/PAM/LiCl/PDA | 0 | 0 |
|  | Negative control | 0 | 0 |
| 72 h | Positive control | 3 | 3 |
|  | PVA/PAM/LiCl/PDA/LiH | 0 | 0 |
|  | PVA/PAM/LiCl/PDA | 0 | 0 |
|  | Negative control | 0 | 0 |
| 120 h | Positive control | 4 | 4 |
|  | PVA/PAM/LiCl/PDA/LiH | 0 | 0 |
|  | PVA/PAM/LiCl/PDA | 0 | 0 |
|  | Negative control | 0 | 0 |

**Reference**

1. Singh VK, Anis A, Banerjee I, Pramanik K, Bhattacharya MK, Pal K. Preparation and characterization of novel carbopol based bigels for topical delivery of metronidazole for the treatment of bacterial vaginosis. *Mater Sci Eng C Mater Biol Appl.* 2014; 44: 151-158.

2. Zhao Z, Zhang B, Chu H, Liang L, Chen B, Zheng H, Guo X. A high-dosage microneedle for programmable lidocaine delivery and enhanced local long-lasting analgesia. *Biomater Adv.* 2022; 133: 112620.

3. Pogatzki EM, Raja SN. A mouse model of incisional pain. *Anesthesiol.* 2003; 99(4): 1023-1027.

4. Xu T, Zhang X, OuY H, Li Z, Liu C, Huang Z, Xu J, Wei J, Nie B, Ma C, et al. Epigenetic upregulation of cxcl12 expression mediates antitubulin chemotherapeutics–induced neuropathic pain. *Pain.* 2017; 158(4): 637-648.

5 Brennan TJ, Vandermeulen EP, Gebhart GF. Characterization of a rat model of incisional pain. *Pain.* 1996; 64(3): 493-502.

6. Zhou B, Yuan W. Tunable thermoresponsive and stretchable hydrogel sensor based on hydroxypropyl cellulose for human motion/health detection, visual signal transmission and information encryption. *Carbohydr Polym.* 2024; 343: 122497.

7. Zhang H, Yang Q, Xu L, Li N, Tan H, Du J, Yu M, Xu J. Triboelectric nanogenerators based on hydrated lithium ions incorporated double-network hydrogels for biomechanical sensing and energy harvesting at low temperature. *Nano Energy.* 2024; 125: 109521.

8. Han S, Tan H, Wei J, Yuan H, Li S, Yang P, Mi H, Liu C, Shen C. Surface modification of super arborized silica for flexible and wearable ultrafast‐response strain sensors with low hysteresis. *Adv Sci.* 2023; 10(25): 2301713.

9. Cai H, Zhang D, Zhang H, Tang M, Xu Z, Xia H, Li K, Wang J. Trehalose-enhanced ionic conductive hydrogels with extreme stretchability, self-adhesive and anti-freezing abilities for both flexible strain sensor and all-solid-state supercapacitor. *Chem Eng J.* 2023; 472: 144849.

10. Jia L, Jiang J, Ren A, Wei Z, Xiang T, Zhou S. Ultra-fast cryogenic self-healing ionic hydrogel for flexible wearable bioelectronics. *Chem Eng J.* 2024; 495: 153734.

11. Roy A, Zenker S, Jain S, Afshari R, Oz Y, Zheng Y, Annabi N. A highly stretchable, conductive, and transparent bioadhesive hydrogel as a flexible sensor for enhanced real‐time human health monitoring. *Adv Mater.* 2024; 36(35): 2404225.

12. Zhang J, Shen S, Lin R, Huang J, Pu C, Chen P, Duan Q, You X, Xu C, Yan B, et al. Highly stretchable and biocompatible wrinkled nanoclay‐composite hydrogel with enhanced sensing capability for precise detection of myocardial infarction. *Adv Mater.* 2023; 35(9): 2209497.

13. Shen Z, Zhang Z, Zhang N, Li J, Zhou P, Hu F, Rong Y, Lu B, Gu G. High‐stretchability, ultralow‐hysteresis conductingpolymer hydrogel strain sensors for soft machines. *Adv Mater.* 2022; 34(32): 2203650.

14. Su G, Wang N, Liu Y, Zhang R, Li Z, Deng Y, Tang B Z. From fluorescence‐transfer‐lightening‐printing‐assisted conductive adhesive nanocomposite hydrogels toward wearable interactive optical information‐electronic strain sensors. *Adv Mater.* 2024; 36(25): 2400085.

15. Chen Z, Shen T, Zhang M, Xiao X, Wang H, Lu Q, Luo Y, Jin Z, Li C. Tough, anti‐fatigue, self‐adhesive, and anti‐freezing hydrogel electrolytes for dendrite‐free flexible zinc ion batteries and strain sensors. *Adv Funct Mater.* 2024; 34(26): 2314864.
